# Development of a Large-Scale Pathogen Screening Test for the Biosafety Evaluation of Canine Mesenchymal Stem Cells

**DOI:** 10.1186/s12575-023-00226-x

**Published:** 2023-12-14

**Authors:** Emese Pekker, Katalin Priskin, Éva Szabó-Kriston, Bernadett Csányi, Orsolya Buzás-Bereczki, Lili Adorján, Valéria Szukacsov, Lajos Pintér, Miklós Rusvai, Paul Cooper, Endre Kiss-Tóth, Lajos Haracska

**Affiliations:** 1 HCEMM-HUN-REN BRC Mutagenesis and Carcinogenesis Research Group, Institute of Genetics, HUN-REN Biological Research Centre, Szeged, H-6726 Hungary; 2 Doctoral School of Interdisciplinary Medicine, University of Szeged, Korányi fasor 10, Szeged, H-6720 Hungary; 3 Delta Bio 2000 Ltd., Szeged, H-6726 Hungary; 4 Visal Plus Ltd., Szeged, H-6726 Hungary; 5 HUN-REN BRC Mutagenesis and Carcinogenesis Research Group, Institute of Genetics, HUN-REN Biological Research Centre, Szeged, H-6726 Hungary; 6 Vet-Diagnostics Ltd., Szolnok, 5000 Hungary; 7 Assentra Limited, Essex, UK; 8 Division of Clinical Medicine, School of Medicine and Population Health, University of Sheffield, S10 2RX, Sheffield, UK; 9 National Laboratory for Drug Research and Development, Magyar tudósok krt. 2. H-1117, Budapest, Hungary

**Keywords:** Canine, Mesenchymal stem cells, Cell therapy, Pathogen testing, EMA guidelines, Validation

## Abstract

**Background:**

The action of mesenchymal stem cells (MSCs) is the subject of intense research in the field of regenerative medicine, including their potential use in companion animals, such as dogs. To ensure the safety of canine MSC batches for their application in regenerative medicine, a quality control test must be conducted in accordance with Good Manufacturing Practices (GMP). Based on guidance provided by the European Medicines Agency, this study aimed to develop and validate a highly sensitive and robust, nucleic acid-based test panel for the detection of various canine pathogens. Analytical sensitivity, specificity, amplification efficiency, and linearity were evaluated to ensure robust assessment. Additionally, viable spike-in controls were used to control for optimal nucleic acid extraction. The conventional PCR-based and real-time PCR-based pathogen assays were evaluated in a real-life setting, by direct testing MSC batches.

**Results:**

The established nucleic acid-based assays displayed remarkable sensitivity, detecting 100–1 copies/reaction of template DNA. They also exhibited high specificity and efficiency. Moreover, highly effective nucleic acid isolation was confirmed by the sensitive detection of spike-in controls. The detection capacity of our optimized and validated methods was determined by direct pathogen testing of nine MSC batches that displayed unusual phenotypes, such as reduced cell division or other deviating characteristics. Among these MCS batches of uncertain purity, only one tested negative for all pathogens. The direct testing of these samples yielded positive results for important canine pathogens, including tick-borne disease-associated species and viral members of the canine infectious respiratory disease complex (CIRDC). Notably, samples positive for the etiological agents responsible for enteritis (CPV), leptospirosis (*Leptospira interrogans*), and neosporosis (*Neospora caninum*) were also identified. Furthermore, we conducted biosafety evaluation of 12 MSC batches intended for therapeutic application. Eleven MSC batches were found to be free of extraneous agents, and only one tested positive for a specific pathogen, namely, canine parvovirus.

**Conclusion:**

In this study, we established and validated reliable, highly sensitive, and accurate nucleic acid-based testing methods for a broad spectrum of canine pathogens.

**Supplementary Information:**

The online version contains supplementary material available at 10.1186/s12575-023-00226-x.

## Background

Increasing evidence suggests that mesenchymal stem cells (MSC) offer a promising option for tissue regeneration and cell therapy [1]. MSCs have attracted extensive research attention in recent years due to their paracrine [2] and immunosuppressive [3, 4] properties as well as their multi-lineage differentiation potential [5]. MSC-based therapy in veterinary medicine targets a wide spectrum of diseases, with the majority focusing on musculoskeletal conditions. Their effectiveness in reducing pain and inflammation makes them an excellent candidate for cell-based therapies for veterinary regenerative medicine [6].

To comply with Good Manufacturing Practices (GMP) and ensure patient safety, clinical batches of MSC undergo mandatory quality control tests to ensure their purity and sterility [7]. To prevent potential introduction of infective agents into the manufacturing process and downstream application in regenerative medicine, it is necessary to subject all clinical batches of MSC donations to screening procedures for extraneous agents. Guidelines provided by the European Medicines Agency’s (EMA) Committee for Medicinal Products for Veterinary Use (CMPV) facilitate quality and security improvement of cell preparations [8, 9]. These guidance documents are indispensable resources, covering critical biosafety aspects that must be taken into consideration in the case of allogeneic stem cell-based therapies. In addition to the starting material, extraneous agents introduced unintentionally during the manufacturing process should also be considered. During the production process, it is crucial to distinguish between exogenous agents (viruses, bacteria, and protozoa) that originate from the raw material and microbiological contamination, which is unrelated to donor tissue [10].

Diagnostic methods traditionally used for canine pathogens, such as viral cultures, microscopic examinations, and serologic tests, as well as traditional microbiological methods, may not be sufficiently sensitive, are often laborious, and time-consuming [11, 12]. Nucleic acid amplification tests (NAATs) provide more accurate and specific approaches, offering faster, more accurate and more cost-effective detection methods than traditional techniques [13].

To date, there are a limited number of optimized and validated in-house-developed nucleic acid-based diagnostic methods for the biosafety evaluation of canine MSCs. Moreover, currently, there are no available nucleic acid-based testing methods for the safety evaluation of a wide range of canine pathogens. Therefore, we proposed the development and validation of different NAATs for the rapid and reliable identification of a broad spectrum of potential extraneous agents in canine MSC batches, obtained from donated tissue, prior to their use in subsequent veterinary therapeutic applications. Our study aimed to establish a reliable, highly sensitive and efficient nucleic acid-based testing panel for convenient, rapid, and cost-effective detection of extraneous agents in MSC batches intended for veterinary use.

Based on the epidemiological considerations for the Carpathian Basin region, a risk assessment was conducted, taking into account the published guidelines for the detection of potential extraneous agents in veterinary medical products. The pathogens to be investigated were selected following the guidelines published by the Committee for Medicinal Products for Veterinary Use (CVMP) [8]. These include bacterial species such as *Leptospira interrogans*, *Brucella canis*, *Neorickettsia* spp., *Rickettsia* spp., *Borrelia* spp., *Ehrlichia* spp., *Bartonella* spp., *Anaplasma* spp.; viruses such as canine adenovirus 1 (CAV-1), canine herpesvirus 1 (CaHV-1), canine parvovirus (CPV), suid herpesvirus 1 (SuHV-1), canine influenza virus (CIV), canine parainfluenza virus (CPIV), canine distemper virus (CDV), canine coronavirus (CCoV), rabies virus, and parasitic species, e.g., *Leishmania* spp.*, Neospora caninum,* and *Babesia* spp. To assess the potential presence of microbiological contamination during culturing of the cells, *Mycoplasma* spp. were also incorporated in the investigation [14].

## Results

### Spike-in Controls

**Fig. 1:**
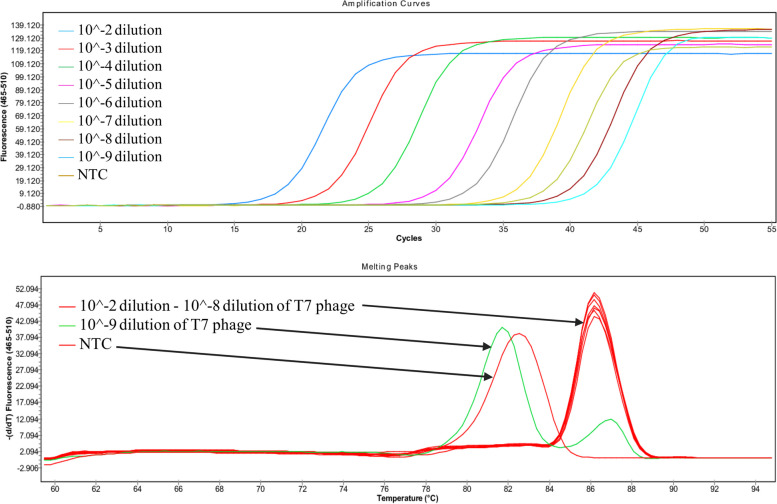
Real-time PCR-based evaluation of T7 bacteriophage spike-in control. **A** Positive amplification of the T7 bacteriophage target sequence using dilution series, **B** HRM results showing specific melting peaks for the target sequences, which are easily distinguishable from the no template control (NTC) and the dilution that does not show a specific signal (10^−9^ dilution of T7 phage)

Optimal nucleic acid extraction was evaluated using exogenous internal controls (IC). The implemented spike-in controls provided evidence of both the amplifiability of DNA and the lack of inhibitors in the samples. Real-time PCR-based assays were used to detect spike-in controls in MSC samples. The real-time PCR assays were able to detect 15 PFU/ml at 10^−8^ dilution of T7 bacteriophage (Fig. 1), the most diluted sample of *Escherichia coli* DH5-alpha (1 CFU/ml; Supplemental Figs. 1 and 2) and MS2 bacteriophage (180 PFU/ml at 10^−8^ dilution; Supplemental Figs. 3 and 4), confirming sensitive detection and ideal nucleic acid extraction. The specific PCR products could be differentiated easily from other products exhibiting distinct melting temperatures. No cross-reactivity, which might hinder interpretation, was observed using the specific primer pairs for the spike-in controls.

The specific PCR products could be differentiated easily from other products exhibiting distinct melting temperatures. No cross-reactivity, which might hinder interpretation, was observed using the specific primer pairs for the spike-in controls.

### Analytical Sensitivity

The limit of detection (LOD) — the lowest copy number of the target at which at least 95% of the samples were considered positive — was assessed using a series of 10-fold dilutions of plasmid DNA containing the target sequence for each pathogen. PCR assays were carried out using a total of 20 replicates of each template DNA concentration. The presence of additional canine DNA had no effect on any of the PCR assays. The LOD for both the conventional PCR-based and the real-time PCR-based assays revealed high analytical sensitivity. The detection limit for DNA and RNA viruses was 10 copies/reaction, except for SuHV-1 and CDV, where the detection limit was 100 copies/reaction, which indicates a sensitive detection of these pathogens using conventional PCR. Protozoa can be also detected with high sensitivity utilizing conventional PCR methods. For *Babesia* spp., the LOD was determined as 100 copies/reaction, whereas the *Neospora* spp. assay showed a detection limit of 10 copies/reaction. Real-time PCR-based assays also displayed high sensitivity. The real-time PCR-based methods employed in this study demonstrated their ability to detect 10 copies/reaction of the selected pathogen, except for *Borrelia* spp., which exhibited a detection limit of 100 copies/reaction. The single-tube real-time TaqMan probe-based Mycoplasma assay showed even higher sensitivity, detecting even 1 copy of the *Mycoplasma* sp. DNA target sequence; see details in Table 1.

**Table 1:** Analytical sensitivity of the assays

Infectious agent		Limit of detection (copies/reaction)	LOD (%)
Bacteria	*Mycoplasma* spp.	1	(20/20) 100%
	*Leptospira interrogans*	100	(20/20) 100%
	*Brucella canis*	100	(20/20) 100%
	*Neorickettsia* spp.	10	(20/20) 100%
	*Rickettsia* spp.	10	(20/20) 100%
	*Borrelia* spp.	100	(20/20) 100%
	*Ehrlichia* spp.	10	(20/20) 100%
	*Bartonella* spp.	10	(20/20) 100%
	*Anaplasma* spp.	10	(20/20) 100%
Protozoa	*Leishmania* spp.	10	(20/20) 100%
	*Neospora* spp.	10	(20/20) 100%
	*Babesia* spp.	100	(20/20) 100%
DNA viruses	CAV-1	10	(19/20) 95%
	CaHV-1	10	(20/20) 100%
	CPV	10	(20/20) 100%
	SuHV-1	100	(20/20) 100%
RNA viruses	CIV	10	(20/20) 100%
	CPIV	10	(20/20) 100%
	CDV	100	(20/20) 100%
	CCoV	10	(19/20) 95%
	Rabies virus	10	(20/20) 100%

### Specificity

**Fig. 2:**
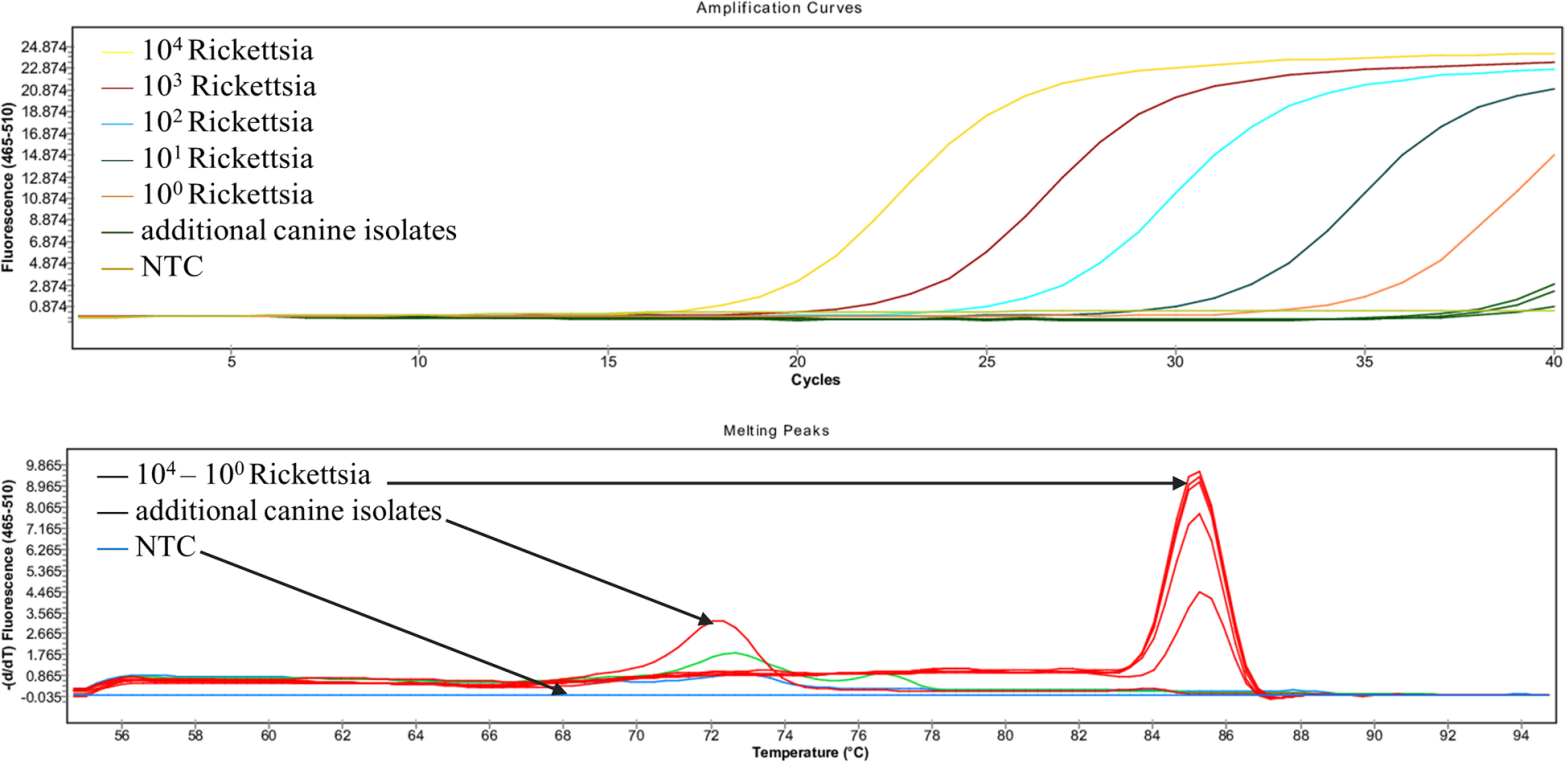
Evaluation of the specificity of the Rickettsia assay. **A** Positive amplification of the target sequence and the three different unamplified canine isolates, **B** HRM results showing specific melting peaks for target sequences are easily distinguishable from the canine isolates containing non-target sequences and the no template control (NTC)

To determine the specificity of each assay, additional canine DNA from three different MSC batches was used in all PCR reactions. For each assay, a serial dilution (10^4^–10^0^ copies/μl) of the pathogen’s positive control plasmid was used as standard along with the additional canine DNA. As shown in Fig. 2, there was no evidence of a false-positive reaction or off-target PCR product with the same melting temperature or size as the expected PCR product. In the Rickettsia assay, canine DNA samples showed no amplification.

### Amplification Efficiency and Linearity

**Fig. 3:**
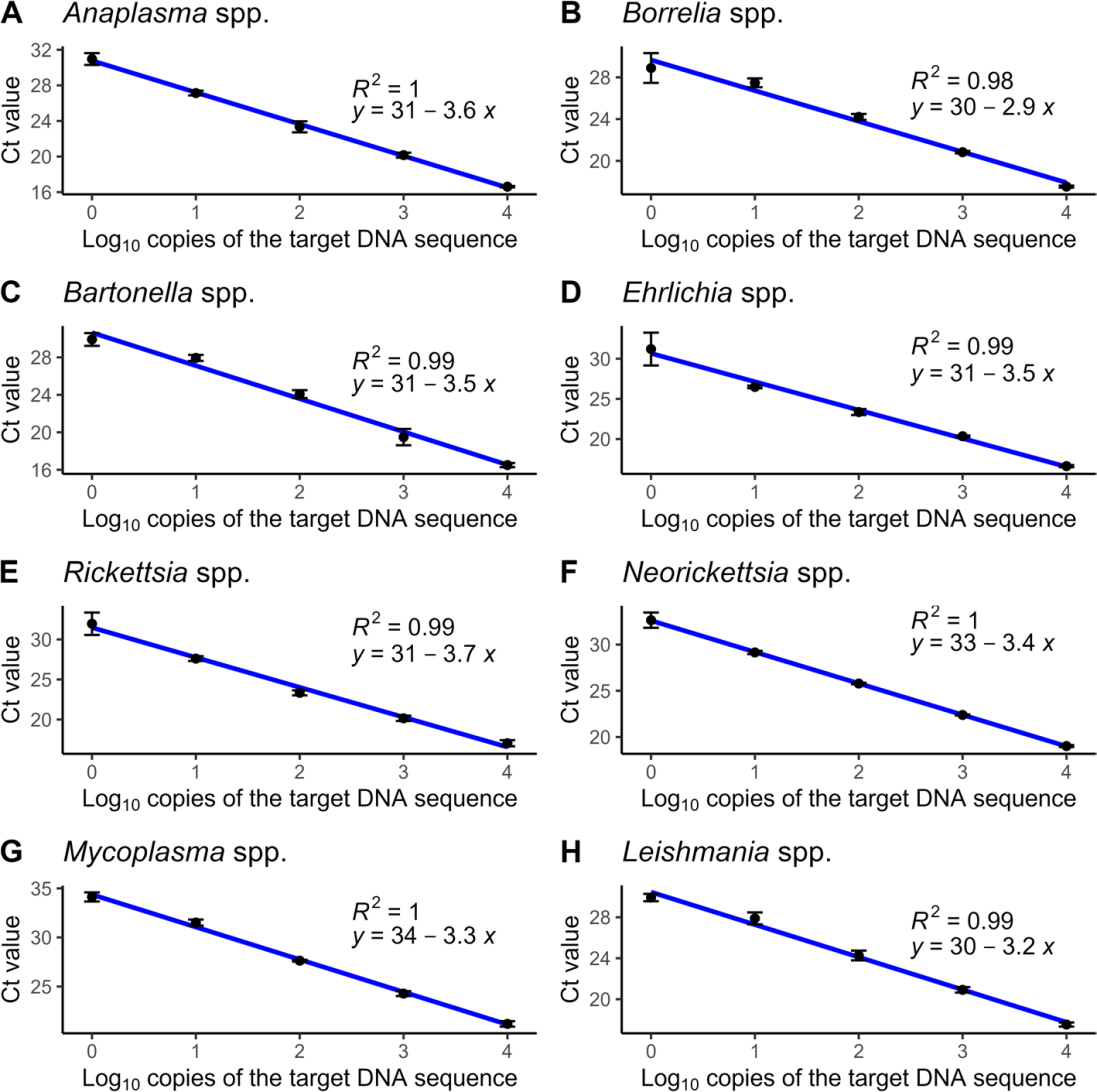
Standard curves showing Ct values plotted against the log10 of the target DNA copy number for **A**. *Anaplasma* spp.; **B**. *Borrelia* spp.; **C**. *Bartonella* spp.; **D**. *Ehrlichia* spp.; **E**. *Rickettsia* spp.; **F**. *Neorickettsia* spp.; *Mycoplasma* spp.; *Leishmania* spp

To determine the amplification efficiency of our real-time PCR-based assays, a serial dilution of the positive control of the relevant pathogen (10^4^–10^0^ copies/μl) was prepared, and each concentration was analyzed in triplicates. In the linear regression analysis, the average Ct values were plotted against the log10 DNA copy number. As shown in Fig. 3, the standard curves of the *Anaplasma* spp., *Borrelia* spp., *Bartonella* spp., *Ehrlichia* spp., *Rickettsia* spp., *Neorickettsia* spp., *Mycoplasma arginini* and *Leishmania* spp. assays revealed that the mean standard curve slopes range from − 3.7 to − 2.9, corresponding to efficiencies between 86.32 and 121.22% (Table 2). Linearity was evaluated for each real-time PCR assay using linear regression analysis by calculating the coefficient of determination (R^2^). The linear regression analysis for the real-time PCR-based assays revealed that R^2^ varied between 0.98 and 1 (Fig. 3).

**Table 2:** Efficiency of the real-time PCR-based assays

Pathogen	*Bartonella* spp.	*Borrelia* spp.	*Rickettsia* spp.	*Neorickettsia* spp.	*Anaplasma* spp.	*Ehrlichia* spp.	*Leishmania* spp.	*Mycoplasma* spp.
**Efficiency**	93.06%	121.22%	86.32%	96.84%	89.57%	93.06%	105.35%	100.92%

### Direct Pathogen Testing of the MSC Batches

The diagnostic efficiency of our nucleic acid-based testing panel for extraneous agents was evaluated by testing different MSC batches. Before nucleic acid extraction, all samples were spiked with our natural spike-in controls: *E. coli* DH5-alpha, MS2 phage, and T7 phage for the extraction of bacterial/protozoan DNA, viral RNA, and viral DNA, respectively. For each sample, 10^4^ copies/μl of spike-in control was used. The concentrations of isolated bacterial/protozoan DNA were between 3.36 ng/μl and 9.63 ng/μl, while the isolated viral RNA and viral DNA concentrations ranged from 4.29 ng/μl to 12.5 ng/μl and 5.53 ng/μl to 21.9 ng/μl, respectively. All spike-in controls were detected at a Ct value< 35 in all MSC samples.

**Fig. 4:**
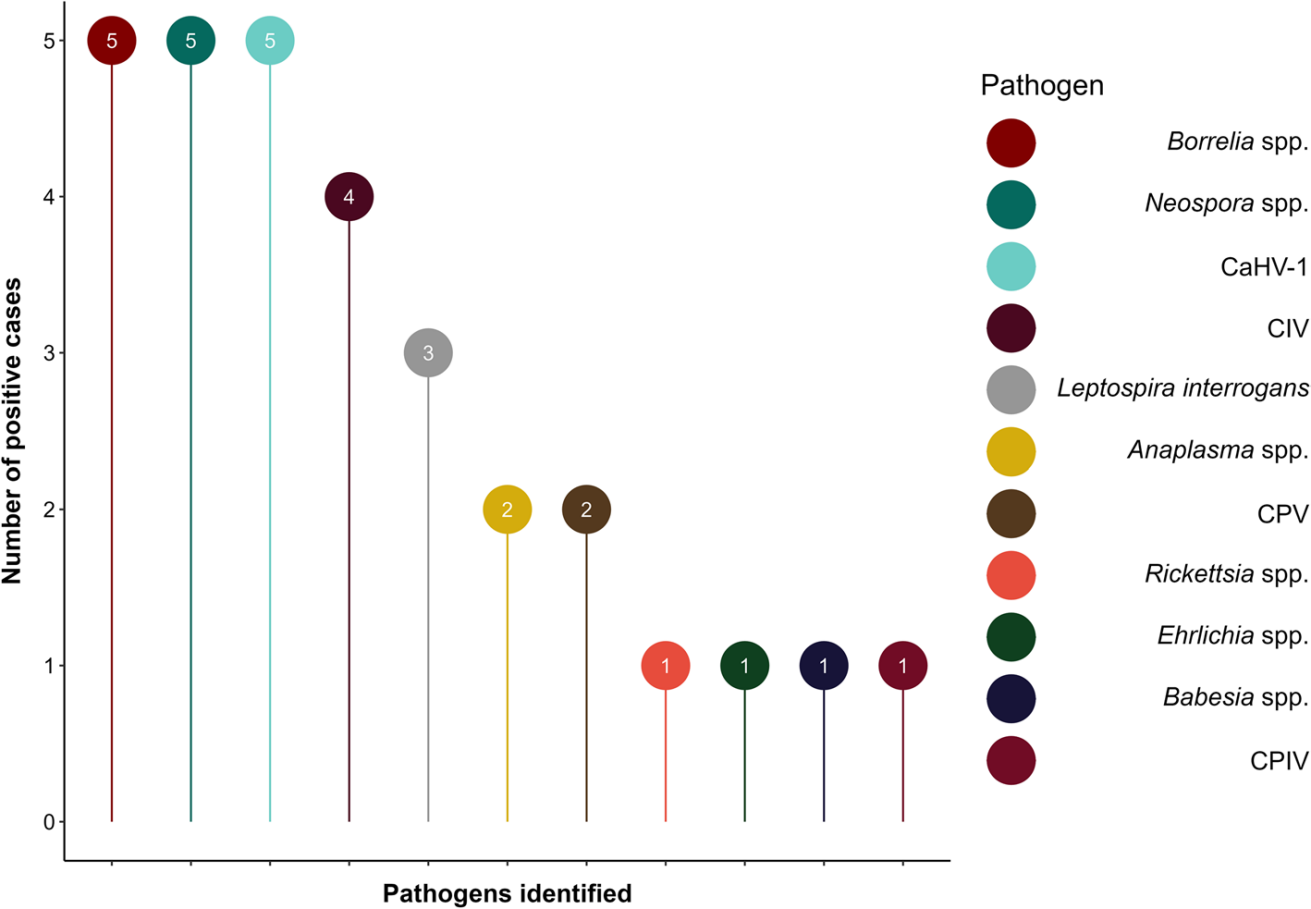
The number of MSC batches that tested positive for *Borrelia* spp., *Neospora* spp., CaHV-1, CIV, *Leptospira interrogans*, *Anaplasma* spp., CPV, *Rickettsia* spp., *Ehrlichia* spp., *Babesia* spp., and CPIV

The effectiveness of our nucleic acid-based pathogen testing methods in detecting a broad range of pathogens was confirmed by direct testing of the nine previously selected samples that were of uncertain purity. Among these MSC samples, only one sample was negative for all the extraneous agents. According to the test results, *Borrelia* spp., *Neospora* spp., and CaHV-1 were the most frequently identified pathogens, which were detected in five positive samples. Four samples were positive for CIV, three for *L. interrogans*, two cases were identified positive for *Anaplasma* spp. and CPV, while only one sample was identified positive for *Rickettsia* spp., *Ehrlichia* spp., *Babesia* spp., and CPIV (Fig. 4). The test results for each extraneous agent are displayed in Table 3.

**Table 3:** Detection of infectious agents in the nine selected samples of substandard MSC quality

Infectious agent	No. of positive samples/No. of total samples
Bacteria	
*Mycoplasma* spp.	0/9
*Leptospira interrogans*	3/9
*Brucella canis*	0/9
*Neorickettsia* spp.	0/9
*Rickettsia* spp.	1/9
*Borrelia* spp.	5/9
*Ehrlichia* spp.	1/9
*Bartonella* spp.	0/9
*Anaplasma* spp.	2/9
Protozoa	
*Leishmania* spp.	0/9
*Neospora* spp.	5/9
*Babesia* spp.	1/9
DNA viruses	
CAV-1	0/9
CaHV-1	5/9
CPV	2/9
SuHV-1	0/9
RNA viruses	
CIV	4/9
CPIV	1/9
CDV	0/9
CCoV	0/9
Rabies virus	0/9

**Fig. 5:**
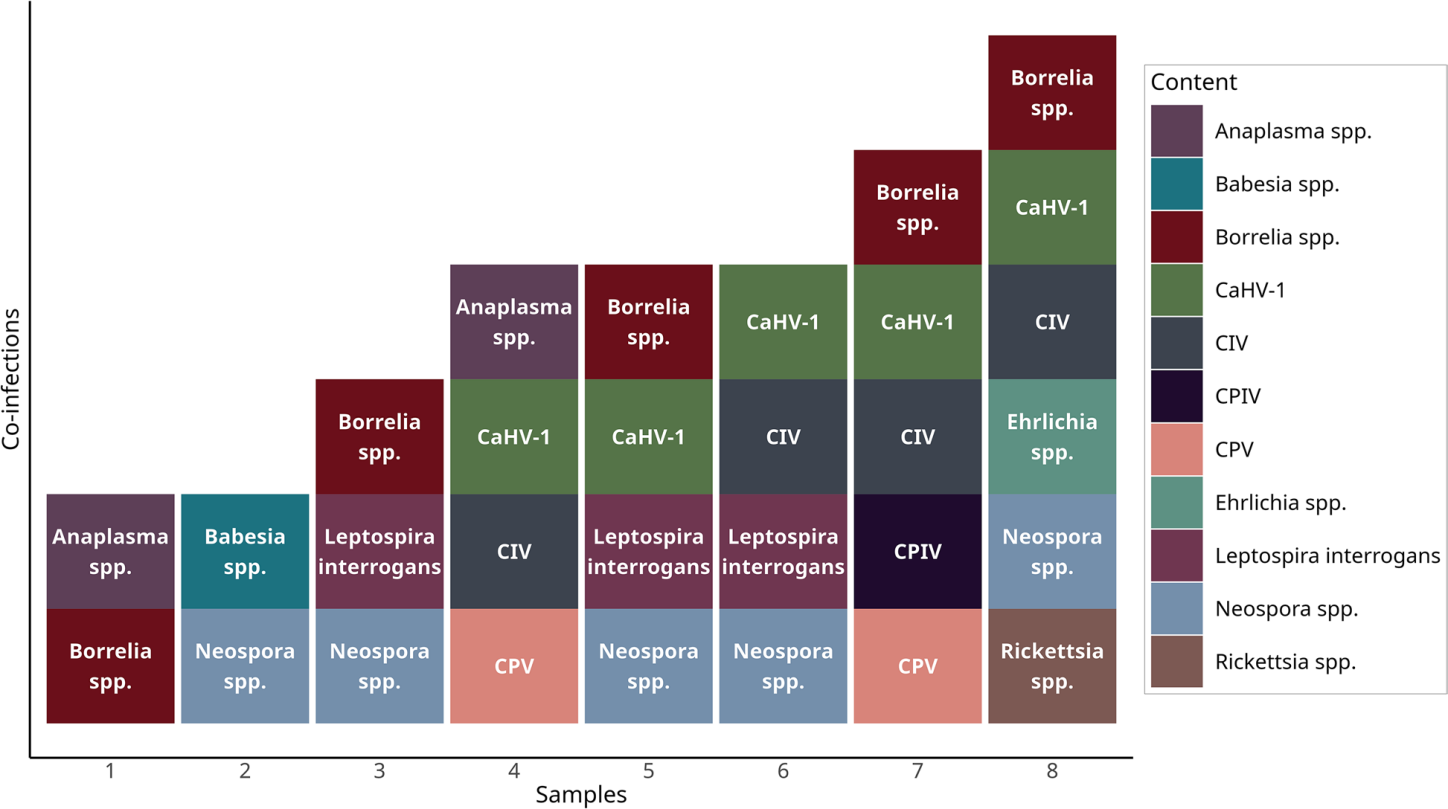
Co-infections detected in the eight positive MSC batches

Among the positive MSC batches, all presented co-infections, testing positive for more than one extraneous agent. We identified a co-infection involving the protozoan parasites *Neospora* spp. and *Babesia* spp. The other dual infection was represented by *Anaplasma* spp. and *Borrelia* spp. We also identified co-infections with multiple extraneous agents. The most common pathogen among the co-infections was CaHV-1, which accounted for 62.5% of the cases (5/8). It was identified in all co-infections involving four or more pathogens (details are shown in Fig. 5).

There were no positive results for *Mycoplasma* spp., *Bartonella* spp., *Neorickettsia* spp., *B. canis*, *Leishmania* spp., CAV-1, SuHV-1, CDV, CCoV or Rabies virus in any of the nine MSC samples (Table 3).

Finally, using the above developed and validated pathogen testing system, we carried out a biosafety evaluation of 12 MSC batches that showed expected proliferative potential and morphology, intended for therapeutic purposes. The analysis of these indicated that only a single sample tested positive for a pathogen, namely, CPV.

## Discussion

Due to their multi-lineage differentiation and immunomodulatory potential, MSCs are outstanding candidates for cell-based therapies in regenerative medicine [15–17]. Recent studies also demonstrated the efficacy of MSC-based therapy in veterinary applications for dogs [18, 19]. The purity and sterility of clinical MSC batches are ensured through quality control testing following Good Manufacturing Practices (GMP) guidelines.

In the present study, epidemiological perspective was employed to select several potential extraneous agents for biosafety evaluation of the MSC batches. A substantial number of potential pathogens were chosen, following the guidelines provided by the European Medicines Agency’s (EMA) Committee for Medicinal Products for Veterinary Use (CMPV). Following a comprehensive literature review, target sequences, primers, and probes for each pathogen were adapted from previously described methods to develop in-house nucleic acid-based testing assays. Optimization of the reaction conditions was performed to achieve a less laborious, highly sensitive, and specific detection approach. To this end, certain pathogen groups were compiled for the implementation of the same run protocol. PCR was performed parallelly with the same protocol for *L. interrogans* and *B. canis;* for *Neorickettsia* spp., *Rickettsia* spp., *Borrelia* spp., *Ehrlichia* spp., *Bartonella* spp., *Anaplasma* spp. and *Leishmania* spp.; for DNA viruses (CAV-1, CaHV-1, CPV and SuHV-1); and for several RNA viruses (CIV, CPIV and CDV). This approach allowed for a less laborious and standardized detection of these pathogens.

The real-time PCR-based detection of the three different natural exogenous spike-in controls was highly consistent between the previously spiked MSC samples. The sensitive detection of the spike-in controls revealed highly efficient nucleic acid isolation. Moreover, the obtained results confirmed the presence of amplifiable DNA at low concentrations and the absence of any PCR inhibitors, emphasizing the significance of these assays in detecting low-copy-number pathogens in MSC batches.

Our recently designed assays showed high efficiency and sensitivity; the LOD for each pathogen ranged from 100 copies/reaction to 1 copy/reaction. Since canine MSC batches may contain low concentrations of extraneous agents, the ability to detect low copy numbers is particularly important. We were able to detect even lower concentrations of the selected pathogens — 10 and 1 copies — though with a significantly lower positive reaction rate, ranging from 25 to 85%, while the 10-fold dilutions above the defined detection limit yielded 100% positive detection in all cases (Table 4).

**Table 4:** Detection rates for the three concentrations of the target pathogens

Infectious agent		Target copies/reaction	Detection rate (%)	Target copies/reaction	Detection rate (%)	Target copies/reaction	Detection rate (%)
Bacteria	*Mycoplasma* spp.	100	(20/20) 100%	10	(20/20) 100%	**1**	(20/20) 100%
	*Leptospira interrogans*	**100**	(20/20) 100%	10	(16/20) 80%	1	(5/20) 25%
	*Brucella canis*	1000	(20/20) 100%	**100**	(20/20) 100%	10	(11/20) 55%
	*Neorickettsia* spp.	100	(20/20) 100%	**10**	(20/20) 100%	1	(5/20) 25%
	*Rickettsia* spp.	100	(20/20) 100%	**10**	(20/20) 100%	1	(16/20) 80%
	*Borrelia* spp.	1000	(20/20) 100%	**100**	(20/20) 100%	10	(15/20) 75%
	*Ehrlichia* spp.	100	(20/20) 100%	**10**	(20/20) 100%	10	(14/20) 70%
	*Bartonella* spp.	100	(20/20) 100%	**10**	(20/20) 100%	1	(7/20) 35%
	*Anaplasma* spp.	100	(20/20) 100%	**10**	(20/20) 100%	1	(14/20) 70%
Protozoan	*Leishmania* spp.	100	(20/20) 100%	**10**	(20/20) 100%	1	(9/20) 45%
	*Neospora* spp.	100	(20/20) 100%	**10**	(20/20) 100%	1	(11/20) 55%
	*Babesia* spp.	1000	(20/20) 100%	**100**	(20/20) 100%	10	(15/20) 75%
DNA viruses	CAV-1	100	(20/20) 100%	**10**	(19/20) 95%	1	(5/20) 25%
	CaHV-1	100	(20/20) 100%	**10**	(20/20) 100%	1	(14/20) 70%
	CPV	100	(20/20) 100%	**10**	(20/20) 100%	1	(7/20) 35%
	SuHV-1	1000	(20/20) 100%	**100**	(20/20) 100%	10	(13/20) 65%
RNA viruses	CIV	100	(20/20) 100%	**10**	(20/20) 100%	1	(10/20) 50%
	CPIV	1000	(20/20) 100%	100	(20/20) 100%	**10**	(20/20) 100%
	CDV	1000	(20/20) 100%	**100**	(20/20) 100%	10	(17/20) 85%
	CCoV	100	(20/20) 100%	**10**	(19/20) 95%	1	(7/20) 35%
	Rabies virus	100	(20/20) 100%	**10**	(20/20) 100%	1	(10/20) 50%

The LOD determined for each pathogen is highlighted in bold

Furthermore, utilizing the internal primers for *L. interrogans*, *B. canis*, *N. caninum,* and canine coronavirus (CCoV) in a single PCR reaction also resulted in high sensitivity. Accordingly, our results are consistent with the previously described detection limit of 100 copies for *L. interrogans* and *B. canis* [20]. In addition, due to the potential cross-reaction of primers with the canine DNA in the same reaction, which could result in false-positive reactions or off-target PCR products, specificity assessments were included. No false-positive results or off-target products that could have influenced the interpretation of the results were detected in any of the specificity assays performed on the three different canine isolates.

In this study, different MSC batches were submitted for pathogen testing. While other testing methods may require a high concentration of starting material for extraneous agent testing, our results showed that the use of 5 × 10^5^ cells and a minimal amount of the corresponding supernatant enabled sensitive and specific detection of the target pathogen. This aspect is significant when applying MSC cells in regenerative medicine, where a considerable number of cells is often required.

Our validated nucleic acid-based assays successfully identified multiple pathogens, including various tick-borne ones, in the nine MSC batches of uncertain purity. Canine tick-borne disease-associated species belong to the genera *Anaplasma*, *Babesia*, *Bartonella*, *Ehrlichia,* and *Rickettsia,* which show an increasing prevalence in different regions of Europe [21–25]. Results of the detection revealed five samples to be positive for *Borrelia* spp., two for *Anaplasma* spp., while only a single sample was detected positive for *Rickettsia* spp., *Ehrlichia* spp. and *Babesia* spp. In contrast, no additional *Bartonella* spp. or hemotropic *Mycoplasma haemocanis* were detected in the specimens. Interestingly, all the positive samples presented at least one pathogen associated with tick-borne diseases. In addition, we identified important viral members of the canine infectious respiratory disease complex (CIRDC). This endemic syndrome involves various viral and bacterial pathogens, which significantly contribute to respiratory illnesses and morbidity in dogs [26, 27]. Among the identified viral members, 5 positive cases were attributed to CaHV-1, 4 positive cases to CIV, and only one sample to CPIV. Furthermore, the present study identified positive samples of etiological agents responsible for enteritis (CPV) in 2 cases, leptospirosis (*L. interrogans*) in 3 cases, and neosporosis (*N. caninum*) in 5 cases. These results are consistent with previous studies on the distribution of these pathogens in Europe; however, they highlight the variability of the epidemiological patterns of these agents across this region [28–30]. All the positive samples showed co-infections. Among the multiple pathogens, CaHV-1 was found to be the predominant agent. None of the nine samples tested positive for *Mycoplasma* spp., *Bartonella* spp., *Neorickettsia* spp., *B. canis*, *Leishmania* spp., CAV-1, SuHV-1, CDV, CCoV, or Rabies virus. In addition, our results obtained from direct pathogen testing of the 12 pre-selected MSC batches for treatment showed that there was only one sample with a positive test result — for Canine Parvovirus (CPV). MSC batches that test positive for any of the pathogens are to be destroyed. One potential limitation of this approach is the inability to distinguish between viable and non-viable pathogens. However, our validated large-scale pathogen testing assays have proved to provide reliable, sensitive, and effective diagnostic performance.

## Conclusions

Compared to traditional microbiological and serological assays, our validated nucleic acid-based detection methods provide a less laborious and reliable diagnosis of a broad spectrum of canine pathogens, exhibiting high sensitivity, specificity, and efficiency. These assays were able to quantify several important canine extraneous agents that were present in MSC batches of uncertain purity, highlighting the great importance of testing. Ultimately, these nucleic acid-based assays — by providing accurate detection of a broad spectrum of canine pathogens — can serve as useful tools for a more comprehensive biosafety evaluation of the MSC batches intended for veterinary use.

## Materials and Methods

### Positive Control Preparation

Positive controls — represented by target-sequence-containing plasmids — were used to optimize and validate each pathogen testing assay. All plasmids contained the target sequence for the pathogen of interest with the primer and probe-binding sites. These plasmids containing the artificial DNA fragments for each pathogen were synthesized by Twist Bioscience (San Francisco, CA), except for the *Mycoplasma* species, which were all cloned in the pBluescript plasmid. All the synthesized plasmids were sequenced to confirm the presence of the specific target sequence. DNA concentration was quantified using Qubit® 3.0 Fluorometer (Invitrogen) and the Qubit® dsDNA HS Assay Kit (Life Technologies), and the DNA copy number was calculated using the following formula:

$$\textrm{Number}\ \textrm{of}\ \textrm{copies}=\frac{\textrm{amount}\ \textrm{of}\ \textrm{dsDNA}\ \textrm{in}\ \textrm{nanograms}\times 6.022\times {10}^{23}\kern0.5em }{\textrm{length}\ \textrm{of}\ \textrm{dsDNA}\ \left(\textrm{bp}\right)\times 1\ \textrm{x}\ {10}^9\times \kern0.5em 660}$$

### Sample Collection and Direct Pathogen Testing of the MSC Batches

MSCs were extracted from visceral adipose tissue acquired as surgical waste, donated by the dogs’ owner via informed consent, following standard ovariectomy of clinically healthy female mixed-breed dogs. The isolated cells were cultured and harvested according to the methodology previously described by Kriston-Pál et al. [31]. To assess the efficacy of our nucleic acid-based pathogen testing methods in detecting the specified pathogens, we separately obtained samples from dog owners and rescue centres that may contain extraneous agents. For this purpose, we processed tissues that did not necessarily meet the originally established isolation criteria, and — monitoring the isolated cells in culture — searched for MSC batches that displayed any abnormal phenotypes such as reduced cell division or altered cell morphology and viability. We collected both supernatants and cell pellets from these samples for further examination. A total of nine MSC batches of uncertain purity were chosen. Furthermore, biosafety investigation was carried out on MSC batches intended for therapeutic utilization. Our biosafety study enrolled 12 batches of MSC batches previously selected for pilot production with the view to use in subsequent treatment.

**Fig. 6:**
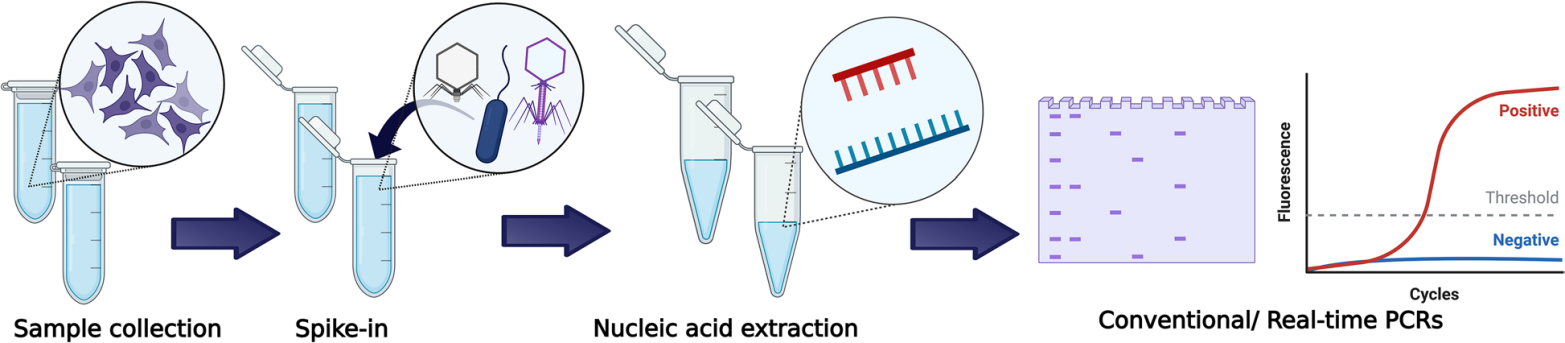
Schematic representation of the pathogen testing workflow. Image created with BioRender.com

Exogenous, natural spike-in controls were used as indicators of optimal nucleic acid extraction. In this context, cells from each sample were previously spiked with viable T7 (single-stranded DNA control) and MS2 (RNA control) bacteriophage and *E. coli* DH5-alpha (double-stranded DNA control). Following spike-in experiments, nucleic acid extraction was carried out for protozoan and bacterial DNA and, separately, total nucleic acid extraction was performed for viruses. Conventional and real-time PCR assays were conducted using the isolated DNA samples, whereas viral RNA was subjected to reverse transcription to produce complementary DNA prior to its inclusion in the PCR reactions. The PCR reactions were performed first to verify the presence and yield from the spike-in controls and then to test for the specific pathogens. The generalized workflow of the pathogen testing procedure is illustrated schematically in Fig. 6.

### Spike-in Controls

As indicators of optimal nucleic acid extraction, exogenous IC were used. These IC provide confirmation of the presence of amplifiable DNA and the absence of inhibitors in the sample. Exogenous heterologous IC were utilized with primers and target sequences that were different from those used for pathogens (Supplemental Tables 1, 2 and 3), ensuring non-competitiveness and ease of implementation. We used natural DNA and RNA as external spike-in controls to control nucleic acid extraction: viable *E. coli* DH5-alpha, MS2 phage, and T7 phage were used as spike-in controls for bacterial/protozoan DNA, viral RNA, and viral DNA extraction, respectively. MS2 bacteriophage (15597-B1TM), T7 bacteriophage (BAA-1025-B2), the appropriate hosts, and *E. coli* DH5-alpha were obtained from the American Type Culture Collection (ATCC). The standardized amount of 5 × 10^5^ MSC samples were spiked with serial dilutions of each spike-in control. These spike-in controls were diluted in parallel to quantify colony forming units (CFUs) for bacteria and plaque forming units (PFUs) for viruses using traditional plating and double-agar overlay methods, respectively. Following nucleic acid extraction from the spiked samples, real-time PCR assays were performed to validate the presence and efficacy of spike-in controls.

### Viral Nucleic Acid Extraction, Quantification, and cDNA Synthesis

Total viral nucleic acid from stem cell samples was extracted using 5 × 10^5^ cells/MSC batch and additionally 100 μl of the corresponding cell supernatant in the case of the nine batches of uncertain purity. Isolation was performed using the MagCore® Viral Nucleic Acid Extraction Kit (RBC Bioscience, Taiwan) following the manufacturer’s recommendation. The final sample was eluted in 100 μl elution buffer. RNA concentration was measured using the Qubit™ RNA High Sensitivity Assay Kit (Invitrogen by Thermo Fisher Scientific), while the concentration of the DNA was measured using the Qubit™ dsDNA High Sensitivity Assay Kit (Invitrogen by Thermo Fisher Scientific) with a Qubit™ 3.0 fluorometer. Following total nucleic acid extraction, High-Capacity cDNA Reverse Transcription Kit (Applied Biosystems) was utilized to generate cDNA for the detection of RNA viruses. Eluted nucleic acids and synthesized cDNA were stored at − 20 °C.

### Bacterial and Protozoan DNA Extraction and Quantification

For bacterial and protozoan DNA extraction, we used the QIAamp® PowerFecal® Pro DNA Kit (QIAGEN®) according to the manufacturer’s protocol. DNA was isolated using 5 × 10^5^ cells/MSC batch and additionally 100 μl of the corresponding cell supernatant in the case of the nine batches of uncertain purity and for *Mycoplasma* detection in all cases. The final sample was eluted in 100 μl elution buffer. Following DNA extraction, a Qubit™ 3.0 fluorometer was used to measure DNA concentration utilizing the associated Qubit™ dsDNA High Sensitivity assay kit (Invitrogen by Thermo Fisher Scientific). Eluted DNA was stored at − 20 °C.

### Primers, Probes and Target Sequences

All primers, probes, and target sequences used in this study were retrieved from previous studies (Table 5). NCBI-BLAST (www.ncbi.nlm.nih.gov) was used to compare and retrieve the nucleotide sequences of each target pathogen. Primer sequences and specific PCR product sizes are described in Table 5. All primer sequences for real-time PCR-based assays were utilized in our study as previously described [36–40], with slight modifications in the application of the primers. Primer sequences for conventional PCRs were used as described previously [32–35, 41], except for CCoV, where the forward primer was combined with the nested reverse primer in a single PCR reaction [32]. A single PCR reaction was also performed for *L. interrogans* [20], *B. canis,* and *N. caninum* [42], employing the previously described internal primers that were initially utilized in the nested PCR, resulting in high specificity, while minimizing the laboriousness of the second round PCR.

**Table 5:** Primers and probes used for extraneous agent detection

	*Infectious agent*	*Detection method*	*Primer name*	*Primer sequence (5′-3′)*	*Product size (bp)*	*Reference*
RNA viruses	CDV	Conventional PCR	CDV_N_F768	AACAGRRATTGCTGAGGACYTAT	290	[32]
			CDV_N_R1057	TCCARRRATAACCATGTAYGGTGC		
	CPIV	Conventional PCR	CPIV_N_F428	GCCGTGGAGAGATCAATGCCTAT	186	[32]
			CPIV_N_R614	GCGCAGTCATGCACTTGCAAGT		
	CIV	Conventional PCR	CIV_M_F151	CATGGARTGGCTAAAGACAAGACC	126	[32]
			CIV_M_R276	AGGGCATTTTGGACAAAKCGTCTA		
	Rabies virus	Conventional PCR	JW12-F	ATGTAACACCYCTACAATG	606/582	[33]
			JW6 UNI-R	ARTTVGCRCACATYTTRTG		
			JW 10 UNI-R	GTCATYARWGTRTGRTGYTC		
	CCoV	Conventional PCR	CoV_16053_F	GGTTGGGAYTAYCCTAARTGTGA	452	[32]
			CoV_Pan_16510_R	TTATARCAVACAACNCCATCATCA		
DNA viruses	CAV-1	Conventional PCR	CadV_E3_F25073	TATTCCAGACTCTTACCAAGAGG	545	[32]
			CadV_E3_R25623	ATAGACAAGGTAGTARTGYTCAG		
	CPV	Conventional PCR	CPV-F	AAGACGTGCAAGCGAGTCC	337	[34]
			CPV-R	GAGCGAAGATAAGCAGCGTAA		
	CaHV-1	Conventional PCR	CaHV_GBF439	ACAGAGTTGATTGATAGAAGAGGTATG	136	[32]
			CaHV_GBR574	CTGGTGTATTAAACTTTGAAGGCTTTA		
	SuHV1	Conventional PCR	gB-Taq-F	CTCCTGCCGCACCTGAAG	92	[35]
			gB-Taq-R	GTCTGGAAGCGGTAGAAGCC		
Bacteria	*Leptospira interrogans*	Conventional PCR	LepN-1	CTGGCCTAAAACTGACGCTGA	171	[20]
			LepN-2	CTTTCACTCTTGCGAGCATAG		
	Borrelia	Real-time PCR	BorP66F	GCAATTTTAGCATCTTTTGGAG	106	[36]
			BorP66R	GATCTATTCCAAAATCRGTWCC		
	*Brucella canis*	Conventional PCR	B2N-1	GTCGCGGATTCTACCTCACCT	281	[20]
			B2N-2	TAAGCAGGTAAGAGGCAATTT		
	*Ehrlichia* spp.	Real-time PCR	Ehr16SF	GGCACGTAGGTGGACTA	101	[36]
			Ehr16SR	TTCCGCTATCCTCTTTCGAC		
	Anaplasma	Real-time PCR	Ana/Ehr16SF	ACACATGCAAGTCGAACG	145	[36]
			Ana/Ehr16SR	CCCCCGCAGGGATTATACA		
	Neorickettsia	Real-time PCR	groel-1500F	ATAGATCCAGCKAAGGTAGTGCGTGT	148	[37]
			groel-1620R	TTCCACCCATGCCACCACCAGGCATCATTG		
	Rickettsia	Real-time PCR	Ric16SF	TCCTAGTGTAGAGGTGAAATTCTTA	178	[36]
			Ric16SR	GAAACCGAAAGAGAATCTTCCGAT		
	Bartonella	Real-time PCR	ssrA-F	GCTATGGTAATAAATGGACAATGAAATAA	298	[38]
			ssrA-R	GCTTCTGTTGCCAGGTG		
	Mycoplasma	Real-time PCR	Myco16sQF1	GCAAAGCTATAGAGATATAGTAGAGGT	106/107 and 100 bp for internal control	[39]
			Myco16sQF2	GCRAAGCTATAGARATATAGTGGAGGT		
			Myco16sQF3	GCAATGCTATAGAGATATAGCGGAGGT		
			Myco16sQF4	GCAAAGCTATGGAGACATAGTGGAGGT		
			Myco16sQF5	GCAAAGTTATGGAAACATAATGGAGGT		
			Myco16sQF6	GCGACGCTATAGAAATATAGTTGAGGT		
			Myco16sQF7	GCAAAGGCTTAGAAATAAGTTCGGAGGC		
			Myco16sQF8	GCAAAGCTATAGAAATATAGTAGAGGT		
			Myco16sQR	GTTGCGYTCGTTGCRGGAC		
			Myco16sQProbe	FAM-TGGTGCATGGTTGTC-MGB		
			Myco16sQICProbe	VIC-CACGCCGTAAACGA-MGB		
Protozoa	Leishmania	Real-time PCR	LEISH-2	ACCCCCAGTTTCCCGCC	119	[40]
			LEISH-1	AACTTTTCTGGTCCTCCGGGTAG		
	Babesia	Conventional PCR	B-BM	GAATCTAAACCCTTCCCAGAGTATC	381/408	[41]
			B-BDV	TGACCTAAACCCTCACCAGAGTAAC		
			B-BDV2	TGACCCAAACCCTCACCAGAGTARC		
			B-rev	GCTTTCGCAGTAGTTCGTCTTTA		
	*Neospora caninum*	Conventional PCR	NS2	CATGTGGATATTTTGCA	146	[42]
			NR1	AAACTCCTGGAAGTTAAAG		

### Real-Time PCR-Based Assays

Real-time PCR-based assays were carried out using the Roche LightCycler® 480 system. The primer concentration was optimized for each real-time PCR-based assay (Supplemental Table 4). Single-tube real-time quantitative PCR (qPCR) was used for Mycoplasma assays. An artificial oligonucleotide was employed as IC, which was simultaneously amplified in a single tube and detected by a specific VIC-labelled TaqMan probe. Eight different forward primers, a single FAM-labelled TaqMan probe, and a single reverse primer were used to detect the strains of interest. A primer mixture of the eight forward primers (4 pmol/μl each) and the FAM-labelled TaqMan probe (1.6 pmol/μl) was used. Real-time PCR reaction was performed in 20 μl reaction volume, containing 10 μl Brilliant III Ultra-Fast qPCR Master Mix (Agilent Technologies), the forward primer mixture, 3.5 pmol/μl reverse primer, and 8 pmol/μl VIC-labelled TaqMan probe. For optimization and validation, 1 μl of the *M. arginini* positive control serial dilution was used as template DNA. In the case of pathogen testing, 1 μl of the DNA template was used for the PCR detection, which was run together with the positive control serial dilution. Nuclease-free water was used as negative control for each PCR run.

Real-time quantitative PCR (qPCR) followed by High Resolution Melting Analysis (HRM) was used for *Bartonella* spp., *Neorickettsia* spp., *Rickettsia* spp., *Anaplasma* spp., *Ehrlichia* spp., *Borrelia* spp., and *Leishmania* spp. assays. Real-time qPCR amplification reactions were performed in 20 μl reaction volumes containing 10 μl 2x Luna® Universal qPCR Master Mix (NEB), 0.8 μl MgCl_2_ (25 mM, Promega), and 0.4–2.5 μl primers (Supplemental Table 4). For optimization and validation, 1 μl of the positive control serial dilution was used as template DNA. In the case of pathogen testing, 1 μl of the DNA template for the PCR detection, which was run together with the positive control serial dilution. Nuclease-free water was used as negative control for each PCR run. Primers and target sequences for real-time PCR-based assays are available in supplemental Tables 5 and 6.

### Conventional PCR-Based Assays

Conventional PCR-based assays were carried out using a Biometra TAdvanced thermocycler (Analytic Jena). Conventional PCR amplification reactions were performed in 25 μl reaction volumes containing 5 μl 5x colourless GoTaq® Flexi buffer (Promega), 2–3 μl MgCl_2_ (25 mM, Promega), 2 μl of 2.5 mM dNTPs (100 mM, Thermo Fisher Scientific), and 0.125 μl of GoTaq® G2 Hot Start Polymerase (Promega, 5 U/μl). The primer concentration was optimized for each assay (Supplemental Table 7). For optimization and validation, 1 μl of the positive control serial dilution was used as template DNA. In the case of direct pathogen testing, 1 μl of the DNA or 1 μl synthesized cDNA was used as template for the PCR detection, which was run together with the positive control serial dilution. Nuclease-free water was used as negative control for each PCR run. Primers and target sequences for conventional PCR-based assays are described separately in Supplemental Tables 8, 9, 10, 11, 12, 13 and 14.

### Analytical Sensitivity

The analytical sensitivity of each assay was evaluated through experimental determination of the LOD, representing the lowest copy number of the target at which at least 95% of the samples give positive signal. LOD was determined by performing a dilution series of the positive control within the anticipated range of detection limits, which was determined during the optimization phase. Three concentrations (1 μl/reaction) of the positive controls were used as follows: expected LOD, 10-fold dilution above the expected LOD, and 10-fold dilution below the expected LOD. Ten replicates were subjected to PCR analysis in two test runs for the three concentrations of the positive control for each pathogen of interest, resulting in 20 independent replicates. A known and standardized concentration of canine DNA (1 ng/μl) isolated from three independent MSC batches, which may potentially influence the detection limit of the assays, was added to each PCR reaction during the sensitivity assessments.

### Specificity

Specificity was evaluated for each pathogen assay by analyzing three different and independent canine DNA isolates within a range of 1–30 ng/μl concentrations. Nuclease-free water was included as negative control in all specificity assays.

### Amplification Efficiency and Linearity

To assess the efficiency of the real-time PCR assays, serial dilutions of the positive control DNA of the relevant pathogen (10^4^–10^0^ copies/μl) were prepared. Each concentration was analysed in triplicate. A linear regression analysis was performed by plotting the average of cycle threshold (Ct) values against the average of the log10 of the corresponding DNA copy number. For each real-time PCR assay, the slope of the regression curve indicates PCR efficiency, which should be between − 3.9 and − 2.9, corresponding to PCR efficiencies ranging from 80% up to 120%. The amplification efficiency was calculated using the following formula: E = [10^(−1/slope)^ − 1] × 100%. Linear regression analysis also allows for the determination of the linearity of our real-time PCR-based assays by the calculation of the coefficient of determination (R^2^).

### Supplementary Information

**Additional file 1: Supplemental Table 1.** Primers and target sequence for the *E. coli* DH5-alpha real-time PCR. **Supplemental Fig. 1.** Real-time PCR results of *E. coli* DH5-alpha serial dilution – stem cell spike-in. **Supplemental Fig. 2.** Real-time PCR results of *E. coli* DH5-alpha dilutions – HRM. **Supplemental Table 2.** Primers and target sequence for the T7 bacteriophage real-time PCR. **Supplemental Table 3.** Primers and target sequence – MS2 phage**. Supplemental Fig. 3.** qPCR results of MS2 phage. **Supplemental Fig. 4.** qPCR results of MS2 phage – HRM**. Supplemental Table 4.** Primer concentrations used in the real-time quantitative PCR-based assays. **Supplemental Table 5.** Primers and target sequences – Mycoplasma assay. **Supplemental Table 6.** Primers and target sequences – real-time PCR-based assays. **Supplemental Table 7.** Primer concentrations used in the conventional PCR-based assays**. Supplemental Table 8.** Primers and target sequences – *Leptospira interrogans* and *Brucella canis*. **Supplemental Table 9.** Primers and target sequences – DNA viruses. **Supplemental Table 10.** Primers and target sequence – Rabies virus. **Supplemental Table 11.** Primers and target sequence – Canine coronavirus**. Supplemental Table 12.** Primers and target sequences – Canine infectious respiratory disease complex (CIRDC) viruses. **Supplemental Table 13.** Primers and target sequence – *N. caninum***. Supplemental Table 14.** Primers and target sequence – Babesia. Informed consent from dog owners.

## Data Availability

All data and materials presented in this study are available upon request (haracska.lajos@brc.hu).

## Abbreviations

MSC: Mesenchymal Stem Cell
GMP: Good Manufacturing Practices
EMA: European Medicines Agency
CMPV: Committee for Medicinal Products for Veterinary Use
NAAT: Nucleic Acid Amplification Test
IC: Internal Control
LOD: Limit of Detection
CT: Cycle Threshold
HRM: High Resolution Melting Analysis
CIRDC: Canine Infectious Respiratory Disease Complex
