# Supplementary material for: Development of a Large-Scale Pathogen Screening Test for the Biosafety Evaluation of Canine Mesenchymal Stem Cells
Source: Biol Proced Online. 2023 Dec 14;25:33. doi: 10.1186/s12575-023-00226-x (PMC10720183; doi:10.1186/s12575-023-00226-x)
Supplement: Supplementary file 1 — Additional file 1: Supplemental Table 1. Primers and target sequence for the E. coli DH5-alpha real-time PCR. Supplemental Fig. 1. Real-time PCR results of E. coli DH5-alpha serial dilution – stem cell spike-in. Supplemental Fig. 2. Real-time PCR results of E. coli DH5-alpha dilutions – HRM. Supplemental Table 2. Primers and target sequence for the T7 bacteriophage real-time PCR. Supplemental Table 3. Primers and target sequence – MS2 phage. Supplemental Fig. 3. qPCR results of MS2 phage. Supplemental Fig. 4. qPCR results of MS2 phage – HRM. Supplemental Table 4. Primer concentrations used in the real-time quantitative PCR-based assays. Supplemental Table 5. Primers and target sequences – Mycoplasma assay. Supplemental Table 6. Primers and target sequences – real-time PCR-based assays. Supplemental Table 7. Primer concentrations used in the conventional PCR-based assays. Supplemental Table 8. Primers and target sequences – Leptospira interrogans and Brucella canis. Supplemental Table 9. Primers and target sequences – DNA viruses. Supplemental Table 10. Primers and target sequence – Rabies virus. Supplemental Table 11. Primers and target sequence – Canine coronavirus. Supplemental Table 12. Primers and target sequences – Canine infectious respiratory disease complex (CIRDC) viruses. Supplemental Table 13. Primers and target sequence – N. caninum. Supplemental Table 14. Primers and target sequence – Babesia. Informed consent from dog owners. [file 12575_2023_226_MOESM1_ESM.docx]

**Supplementary material for ‘Development of a large-scale pathogen screening test for the biosafety evaluation of canine mesenchymal stem cells’**

**Spike-in controls**

**Propagation, enumeration, and PCR detection of spiked *E. coli* DH5-alpha**

*E. coli* DH5-alpha was used as an indicator of optimal bacterial and protozoan nucleic acid extraction. In this context, 5x10^5^ stem cells were spiked using 100 µl of serial dilutions of *E. coli* DH5-alpha bacterial suspension (10^-2^–10^-8^). 100 µl of the dilution series of viable bacteria was also plated on agar plates and grown overnight to measure viable bacteria for spike-in experiments by calculating Colony Forming Units (CFU), considering only countable colonies. Following enumeration, nucleic acid from stem cell samples with *E. coli* DH5-alpha spike-in was isolated using the QIAamp® PowerFecal® Pro DNA Kit (QIAGEN®), following the manufacturer’s recommendations.

Real-time PCR was used to detect spiked *E. coli* DH5-alpha in samples. *E. coli* DH5-alpha primers amplified a 113-bp sequence, the single-copy chromosomal gene d-1-deoxyxylulose-5-phosphate synthase (dxs); details are shown in Supplemental Table 1.

**Supplemental Table 1.** Primers and target sequence for the *E. coli* DH5-alpha real-time PCR assay

| Agent | Primer name and sequence (5’-3’) | Target sequence (5’-3’) | Product size (bp) |
| --- | --- | --- | --- |
| E. coli DH5-alpha | **Forward primer**  CGAGAAACTGGCGATCCTTA | CGAGAAACTGGCGATCCTTAACTTTGGTACGCTGATGCCAGAAGCGGCGAAAGTCGCCGAATCGCTGAACGCCACGCTGGTCGATATGCGTTTTGTGAAACCGCTTGATGAAG | 113 |
|  | **Reverse primer**  CTTCATCAAGCGGTTTCACA |  |  |

Real-time PCR reaction was carried out in a total volume of 25 µl, including 5x colorless GoTaq® Flexi buffer (Promega), 2 µl MgCl_2_ (25 mM, Promega), 2 µl of 2.5 mM dNTPs (100 mM, Thermo Fisher Scientific), 0.5 µl of the forward and reverse primers each (5 pmol/µl), 1.25 µl of EvaGreen dye (Biotium), and 0.125 µl of GoTaq® G2 Hot Start Polymerase (Promega, 5 U/µl). From each spiked sample, 1 µl of eluted DNA was used as template. A dilution series (10^-2^–10^-8^) of *E. coli* DH5-alpha positive control DNA was used as positive control for the PCR reaction. Nuclease-free water was used as negative control for the PCR run.

The PCR running conditions were 95ºC for 2 min followed by 40 cycles of 95ºC for 15 s, 58ºC for 30 s, and 72ºC for 30 s. In addition, a melt curve was run starting from 72ºC followed by 55ºC for 1 s and finally by a stepwise increase in the temperature from 55ºC to 95ºC, with a rate of 0.02 Cº/s. The qPCR reaction was performed using LightCycler® 480 Real-Time PCR System (Roche). High Resolution Melting Analysis (HRM) was used to analyze PCR products.

The real-time PCR assay was able to detect even the most diluted sample (10^-8^) of *E. coli* DH5-alpha (1 CFU/ml), indicating sensitive detection and optimal nucleic acid extraction. The qPCR products can easily be distinguished from other products that melt at different temperatures, in this case a primer dimer in the no template control (NTC).


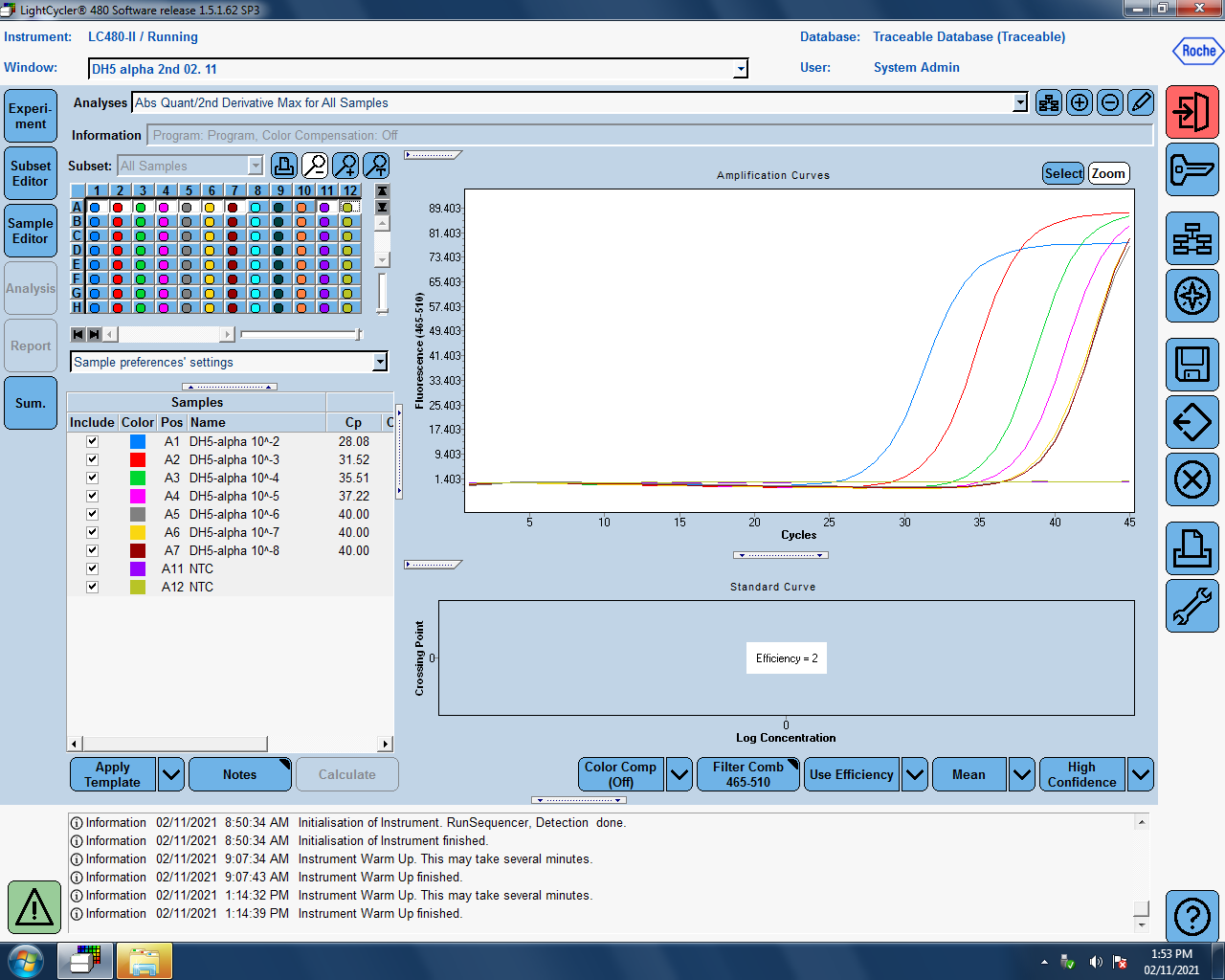


**—** 10^-2^ dilution

**—** 10^-3^ dilution

**—** 10^-4^ dilution

**—** 10^-5^ dilution

**—** 10^-6^ dilution

**—** 10^-7^ dilution

**—** 10^-8^ dilution

— NTC

— NTC

**Supplemental Figure 1** Real-time PCR results of *E. coli* DH5-alpha serial dilution – stem cell spike-in


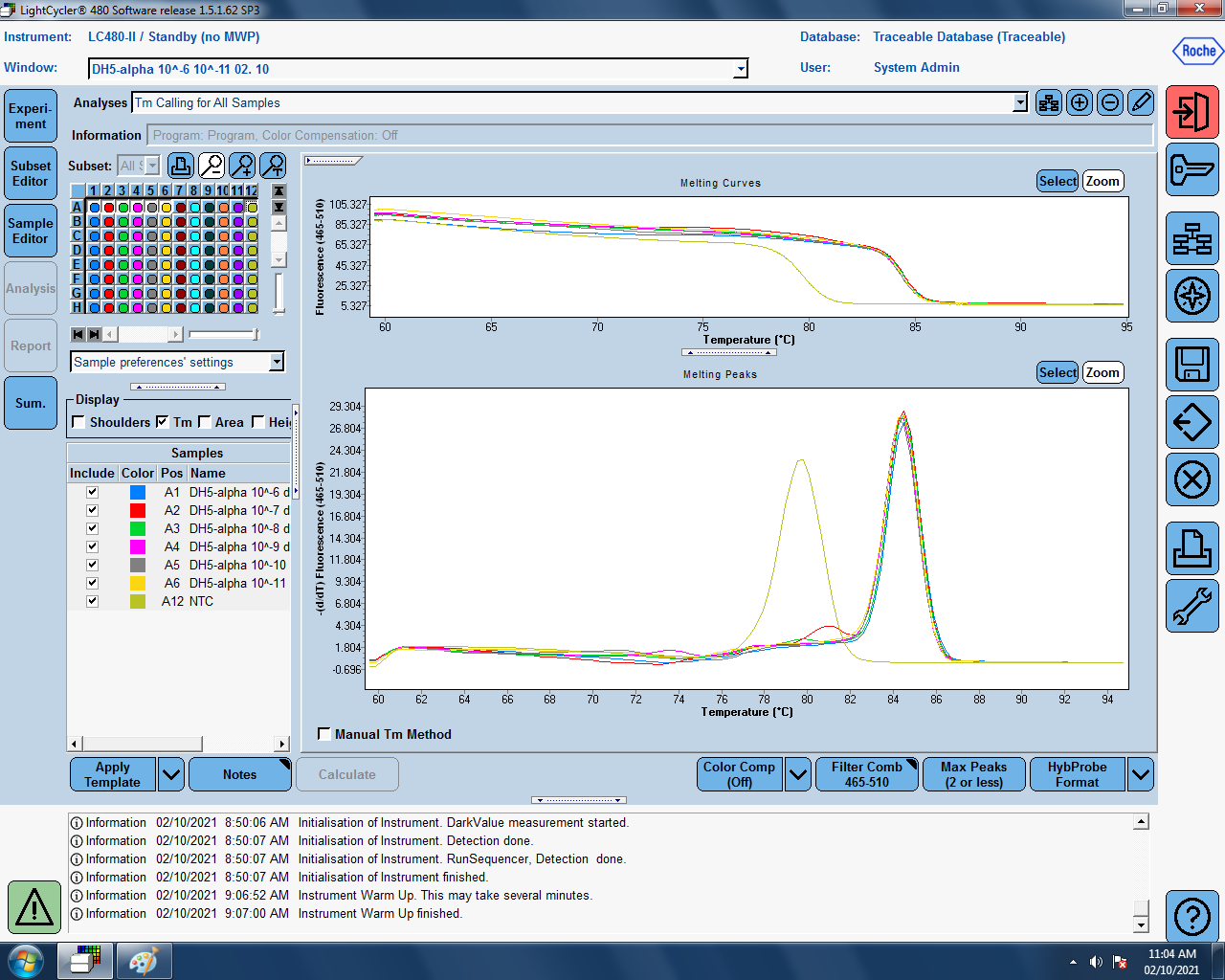


NTC *E. coli* DH5-alpha serial dilution

**Supplemental Figure 2** Real-time PCR results of *E. coli* DH5-alpha dilutions – HRM

**Propagation, enumeration, and PCR detection of spiked T7 bacteriophage**

T7 bacteriophage was used as an indicator of optimal viral DNA extraction. In this context, 5x10^5^ stem cells were spiked using a 100 µl serial dilution of a T7 bacteriophage suspension (10^-2^–10^-9^). Viable T7 was enumerated using the double agar overlay method. For this purpose, 100 µl of each dilution of viable T7 phage was mixed with 100 µl host bacteria (BW25113 *E. coli*) culture and 4.8 ml of 0.5% LB soft agar (at 45°C), plated on agar plates, and grown overnight.

A control plate with the host bacteria BW25113 *E. coli* was included without any T7 phage added to ensure no plaques are formed, indicating no viral contamination. Following enumeration, nucleic acid from stem cell samples with T7 bacteriophage spike-in was isolated using the MagCore® Viral Nucleic Acid Extraction Kit (RBC Bioscience, Taiwan), following the manufacturer’s recommendation.

Real-time PCR was used to detect spiked T7 bacteriophage in stem cell samples. T7 phage primers amplify a 118-bp sequence of the T7 phage major capsid protein (MCP) gene (Supplemental Table 2).

**Supplemental Table 2.** Primers and target sequence for the T7 bacteriophage real-time PCR assay

| Agent | Primer name and sequence (5’-3’) | Target sequence (5’-3’) | Product size (bp) |
| --- | --- | --- | --- |
| Escherichia phage T7 | **Forward primer**  CCGCAACGTTATGGGCTTTG | CCGCAACGTTATGGGCTTTGAGGTTGTAGAAGTTCCGCACCTCACCGCTGGTGGTGCTGGTACCGCTCGTGAGGGCACTACTGGTCAGAAGCACGTCTTCCCTGCCAATAAAGGTGAG | 118 bp |
|  | **Reverse primer**  CTCACCTTTATTGGCAGGGAAG |  |  |

Real-time PCR reaction was carried out in a total volume of 25 µl, including 5x colorless GoTaq® Flexi buffer (Promega), 2 µl MgCl_2_ (25 mM, Promega), 2 µl of 2.5 mM dNTPs (100 mM, Thermo Fisher Scientific), 1 µl of forward and reverse primers each (5 pmol/µl), 1.25 µl of EvaGreen dye (Biotium), and 0.125 µl of GoTaq® G2 Hot Start Polymerase (Promega, 5 U/µl). From each spiked sample, 1 µl of eluted DNA was used as template. A dilution series (10^-2^–10^-9^) of T7 bacteriophage positive control DNA was used as positive control for the PCR reaction. Nuclease-free water was used as negative control for the PCR run.

The PCR running conditions were 95ºC for 2 min followed by 55 cycles of 95ºC for 15 s, 70ºC for 30 s, and 72ºC for 30 s with final extension at 72ºC for 2 min. In addition, a melt curve was run starting from 72ºC followed by 60ºC for 1 s and finally by a stepwise increase in the temperature from 55ºC to 95ºC, with a rate of 0.02 Cº/s. The qPCR reaction was performed using the LightCycler® 480 Real-Time PCR System (Roche). HRM method was used to analyze PCR products. The real-time PCR assay was able to detect the 10^-8^ dilution of T7 bacteriophage (15 PFU /ml), indicating sensitive detection and optimal nucleic acid extraction.

The PCR products can easily be distinguished from other products that melt at different temperatures, in this case a primer dimer in the no template control (NTC) and in the most diluted T7 phage sample (10^-9^ dilution of T7 phage).

**Propagation, enumeration, and PCR detection of spiked MS2 bacteriophage**

MS2 bacteriophage, a single-stranded RNA virus, was used as indicator of optimal viral RNA extraction. Double agar overlay method was used for the propagation and enumeration of bacteriophage MS2. In this context, 5x10^5^ stem cells were spiked with a serial dilution (10^-2^–10^-9^ dilution) of MS2 phage. 100 µl of each dilution of viable MS2 was mixed with 100 µl bacterial host culture and 4.8 ml of 0.5% LB soft agar (at 45°C), plated on agar plates, and grown overnight. Enumeration of viable viruses was assessed by calculating PFU (Plaque-Forming Units). A control plate with host *E. coli* was included to ensure no plaques are formed, indicating no viral contamination. Nucleic acid from stem cell samples with MS2 phage spike-in was then isolated using the MagCore® Viral Nucleic Acid Extraction Kit (High Sensitivity) following the manufacturer’s recommendation.

Real-time PCR was used to detect spiked MS2 bacteriophage in stem cells. After RNA isolation and quantification, cDNA synthesis was carried out using the High-Capacity cDNA Reverse Transcription Kit, following the manufacturer’s recommendations.

MS2 phage primers amplify a 101-bp partial sequence of the MS2 phage genome; details shown in Supplemental Table 3.

**Supplemental Table 3.** Primers and target sequence – MS2 phage

| Agent | Primer name and sequence (5’-3’) | Target sequence (5’-3’) | Product size |
| --- | --- | --- | --- |
| MS2 phage | **Forward primer**  CTCTGAGAGCGGCTCTATTGGT | CTCTGAGAGCGGCTCTATTGGTCCGAGACCAATGTGCGCCGTGGATCAGACACGCGGTCCGCTATAACGAGTCATATGAATTTAGGCTCGTTGTAGGGAAC | 101 bp |
|  | **Reverse primer**  GTTCCCTACAACGAGCCTAAATTC |  |  |

Real-time PCR reaction was carried out in a total volume of 20 µl Master Mix, including 5x Q5® reaction buffer (New England Biolabs), 1.6 µl of 2.5 mM dNTPs (100 mM, Thermo Fisher Scientific), 0.5 µl of forward and reverse primers each (3 pmol/µl), 1.25 µl of EvaGreen dye (Biotium), and 0.2 µl of Q5® High-Fidelity DNA Polymerase (New England Biolabs).

The real-time PCR running conditions were 98ºC for 1 min followed by 40 cycles of 98ºC for 30 s, and 60ºC for 45 s. In addition, a melt curve was run starting from 60ºC followed by 60ºC for 1 s and finally by a stepwise increase in the temperature from 60ºC to 95ºC, with a rate of 0.02 Cº/s. The reaction was performed using the LightCycler® 480 Real-Time PCR System (Roche). HRM method was used to analyze qPCR products.

The qPCR assay was able to detect 180 PFU/ml of MS2 bacteriophage, represented by a 10^-8^ dilution, indicating sensitive detection and optimal nucleic acid extraction. The qPCR products can easily be distinguished from other products that melt at different temperatures (Supplemental Figure 4), in this case a primer dimer in the no template control (NTC).


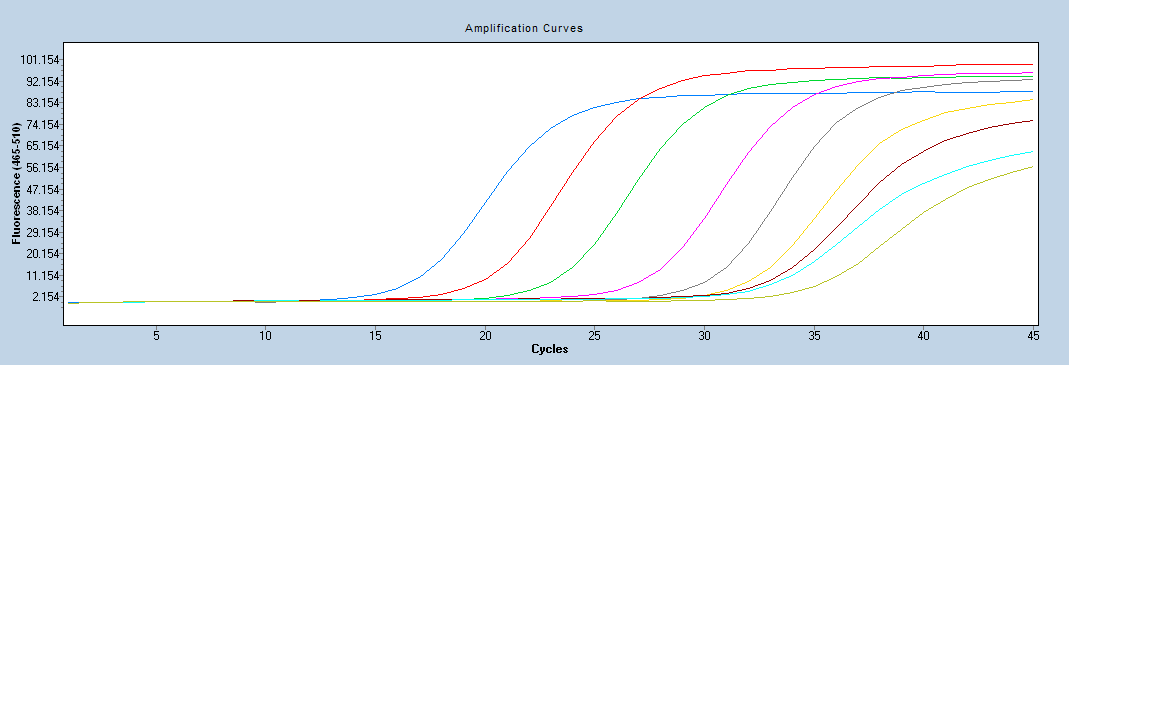


**—** 10^-2^ dilution

**—** 10^-3^ dilution

**—** 10^-4^ dilution

**—** 10^-5^ dilution

**—** 10^-6^ dilution

**—** 10^-7^ dilution

**—** 10^-8^ dilution

**—** 10^-9^ dilution

**—** NTC

**Supplemental Figure 3** qPCR results of MS2 phage


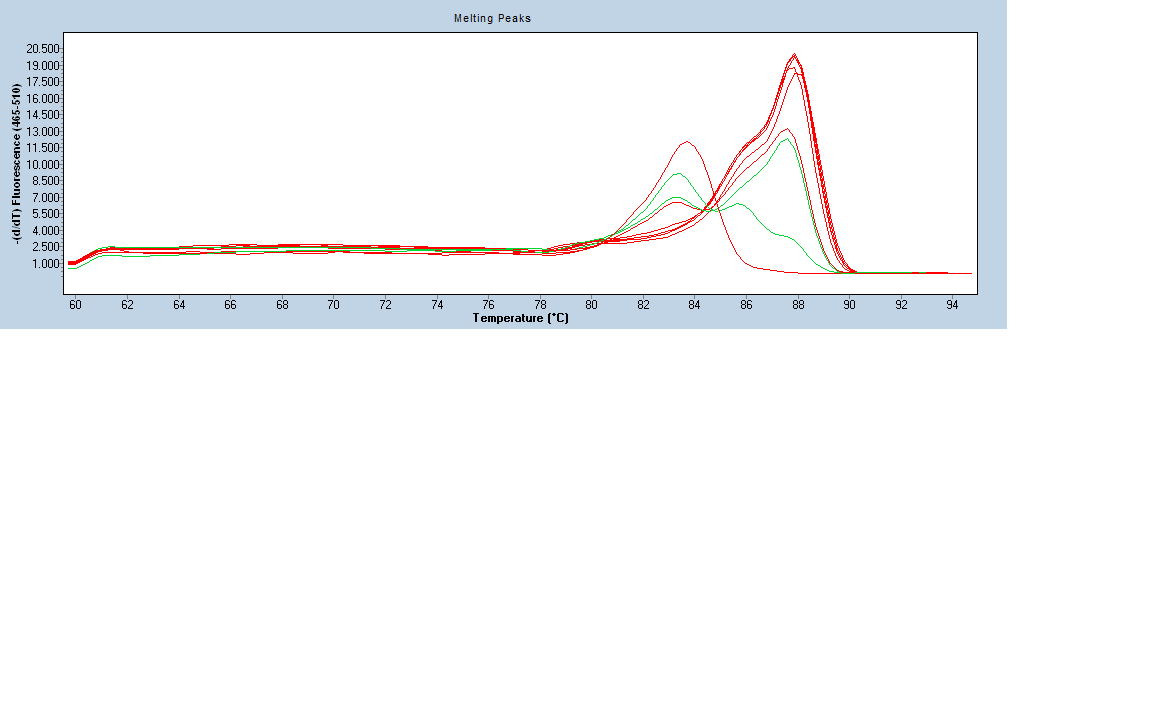


**—** 10^-2^ - 10^-8^ dilution

**—** 10^-9^ dilution

**—** NTC

**Supplemental Figure 4** qPCR results of MS2 phage – HRM

**Real-time PCR-based assays**

Real-time PCR-based assays were performed using the Roche LightCycler® 480 instrument. Primer concentrations were optimized for each real-time PCR-based pathogen assay; see Supplemental Table 4.

**Supplemental Table 4.** Primer concentrations used in the real-time quantitative PCR-based assays

| Infectious agent | | Primer concentration | Amount used  in Master Mix |
| --- | --- | --- | --- |
| Bacteria | Bartonella | 10 pmol/µl | 0.8 µl |
|  | Neorickettsia | 15 pmol/µl | 0.4 µl |
|  | Rickettsia | 20 pmol/µl | 1 µl |
|  | Anaplasma | 25 pmol/µl | 0.8 µl |
|  | Ehrlichia | 15 pmol/µl | 1.2 µl |
|  | Borrelia | 10.5 pmol/µl | 1.2 µl |
|  | Mycoplasma | 4 / 3.5 pmol/µl | 3.125 µl |
| Protozoa | Leishmania | 2 pmol/µl | 2.5 µl |

Mycoplasma detection

Single-tube real-time quantitative PCR (qPCR) was used to detect Mycoplasma species. We used an artificial oligonucleotide as internal control (IC), which was simultaneously amplified in a single tube and detected by a specific VIC-labeled TaqMan probe. Eight different forward primers, a single FAM-labeled TaqMan probe, and a single reverse primer were used to detect the strains of interest.

The PCR reaction mixture (20 µl) contained 10 µl Brilliant III Ultra-Fast qPCR Master Mix (Agilent Technologies), 3.125 µl of the primer pool containing the 8 different forward primers (4 µM each), and 1.6 µM of FAM-labeled TaqMan probe, 3.125 µl of 3.5 µM reverse primer and 0.0625 µl of 8 µM VIC-labeled TaqMan probe. The mixture was made up to the final volume of 18 µl with nuclease-free water. 1 µl of 100 copies of Mycoplasma internal control and 1 µl of isolated DNA was added to the mixture.

The PCR running conditions were 95ºC for 3 min followed by 40 cycles of 95ºC for 10 s and 60ºC for 15 s. The qPCR reaction was performed using the LightCycler® 480 Real-Time PCR System. Specific primers and TaqMan probe (FAM-labeled) were used to detect a 16S rDNA sequence of mollicutes. Primers, probes, target sequences, and internal control sequences are shown in Supplemental Table 5.

**Supplemental Table 5.** Primers and target sequences – Mycoplasma assay

| Primer/probe | Specificity | Target sequence (5’-3’) | Sequence length (base pairs) |
| --- | --- | --- | --- |
| Myco16sQF1  Forward primer  gcaaagctatagagatatagtagaggt | ***Mycoplasma orale, M. mycoides, M. capricolum, and IC*** | GCAAAGCTATAGAGATATAGTGGAGGTTAACAGGGTGACAGATGGTGCATGGTTGTCGTCAGCTCGTGTCGTGAGATGTTTGGTCAAGTCCTGCAACGAGCGCAAC | 106 bp |
| Myco16sQF2  Forward primer  gcraagctatagaratatagtggaggt | ***M. arthritidis, M. gallisepticum, M. hominis, M. hyorhinis, M. penetrans, M. pirum, M. salivarium, M. synoviae*** | GCAAAGCTATAGAGATATAGTGGAGGTTAACAGAATGACAGATGGTGCATGGTTGTCGTCAGCTCGTGTCGTGAGATGTTAGGTTAAGTCCTGCAACGAGCGCAAC | 106 bp |
| Myco16sQF3  Forward primer  gcaatgctatagagatatagcggaggt | ***M. arginine*** | GCAATGCTATAGAGATATAGCGGAGGTTAACGGAGTGACAGATGGTGCATGGTTGTCGTCAGCTCGTGTCGTGAGATGTTTGGTCAAGTCCTGCAACGAGCGCAAC | 106 bp |
| Myco16sQF4  Forward primer  gcaaagctatggagacatagtggaggt | ***M. Fermentans*** | GCAAAGCTATGGAGACATAGTGGAGGTTAACAGAATGACAGATGGTGCATGGTTGTCGTCAGCTCGTGTCGTGAGATGTTTGGTTAAGTCCTGCAACGAGCGCAAC | 106 bp |
| Myco16sQF5  Forward primer  gcaaagttatggaaacataatggaggt | ***M. pneumoniae, M. genitalium*** | GCAAAGTTATGGAAACATAATGGAGGTTAACCGAGTGACAGGTGGTGCATGGTTGTCGTCAGCTCGTGTCGTGAGATGTTGGGTTAAGTCCCGCAACGAGCGCAAC | 106 bp |
| Myco16sQF6  Forward primer gcgacgctatagaaatatagttgaggt | ***Ureaplasma urealyticum*, U. parvum*** | GCGACGCTATAGAAATATAGTTGAGGTTAACAATATGACAGGTGGTGCATGGTTGTCGTCAGCTCGTGTCGTGAGATGTTGGGTTAAGTCCCGCAACGAGCGCAAC | 106 bp |
| Myco16sQF7  Forward primer  gcaaaggcttagaaataagttcggaggc | ***Acholeplasma laidlawii*** | GCAAAGGCTTAGAAATAAGTTCGGAGGCTAACAGATGTACAGGTGGTGCACGGTTGTCGTCAGCTCGTGTCGTGAGATGTTGGGTTAAGTCCCGCAACGAGCGCAAC | 107 bp |
| Myco16sQF8  Forward primer  gcaaagctatagaaatatagtagaggt | ***M. haemocanis*** | GCAAAGCTATAGAAATATAGTAGAGGTTATCGAGGTGACAGGTGGTGCATGGCTGTCGTCAGCTCGTGTCTTGAGATGTTTGGTTAAGTCCCGCAACGAGCGCAAC | 106 bp |
| Myco16sQR  Reverse primer  gttgcgytcgttgcrggac | **Internal control and all target sequences** | GGCAAAGCTATAGAGATATAGTAGAGGTTAGTCCACGCCGTAAACGATATGCTCGCAAGAGTAACCTTCGCAAAGCTATAGTCCTGCAACGAGCGCAACC | 100 bp |
| Myco16sQProbe  FAM-tggtgcatggttgtc-MGB | **All Mycoplasma target sequences** | All the above Mycoplasma target sequences | 15 bp |
| Myco16sQICProbe  VIC-cacgccgtaaacga-MGB | **Internal control** | GGCAAAGCTATAGAGATATAGTAGAGGTTAGTCCACGCCGTAAACGATATGCTCGCAAGAGTAACCTTCGCAAAGCTATAGTCCTGCAACGAGCGCAACC | 100 bp |

Bartonella, Neorickettsia, Rickettsia, Anaplasma, *Ehrlichia* spp*.*, Borrelia, and Leishmania detection

Real-time quantitative PCR (qPCR) followed by the HRM method was performed using 2x Luna® Universal qPCR Master Mix. The PCR reaction mixture (20 µl) contained 10 µl of 2x Luna® Universal qPCR Master Mix, 0.8 µl of 25 mM MgCl_2_ solution (Promega®); for specific concentration and volume of each primer pair used in the mixture, see Table S6. The mixture was made up to the final volume of 19 µl with nuclease-free water, and finally 1 µl of isolated DNA was added to the mixture.

The PCR running conditions were 95ºC for 1 min followed by 40 cycles of 95ºC for 15 s, 58ºC for 30 s, and 72ºC for 30 s. In addition, a melt curve was run starting from 72ºC followed by 55ºC for 1 s and finally by a stepwise increase in the temperature from 55ºC to 95ºC, with a rate of 0.03 Cº/s. The qPCR reaction was performed using the LightCycler® 480 Real-Time PCR System.

Specific primer sequences were used to detect a 297-bp region of the ssrA gene of Bartonella (CP003124.1:757782-758078 *Bartonella vinsonii* subsp. *berkhoffii* str. Winnie, complete genome), a 119-bp fragment of the *Leishmania* kinetoplast DNA minicircle (CACT01000032.1:1284-1402 *Leishmania infantum* JPCM5 WGS CACT00000000 data, contig 38, whole genome shotgun sequence), a 148-bp region of the 3’ end of the heat shock protein-coding gene GroEL of *Neorickettsia* (AY050314.1:1960-2107 *Neorickettsia* sp. SF agent heat shock protein GroES (groES) gene, complete cds; and heat shock protein GroEL gene). Primers Ana/Ehr16SF/R were used to detect the V1-V2 region of the 16S rRNA gene of *Anaplasma* spp. (146 bp), the Ehr16S forward/reverse primers to detect the 16S rRNA V3-V4 region of *Ehrlichia* spp. (101 bp), the Ric16SF/R primers to detect the 16S rRNA V4-V5 region of *Rickettsia* (178 bp), and the BorP66F/R primers to detect the 3′-end of the p66 porin gene of *Borrelia burgdorferi* (106 bp). Primers and target sequences are shown in Supplemental Table 6.

**Supplemental Table 6.** Primers and target sequences – real-time PCR-based assays

| Infectious agent | Primer name and sequence (5’-3’) | Target sequence (5’-3’) | Sequence length (bp) |
| --- | --- | --- | --- |
| Bartonella | **Forward primer**  **ssrA-F** GCTATGGTAATAAATGGACAATGAAATAA | GCTATGGTAATAAATGGACAATGAAATAAGCTTATTGGACCCGGGGGCGGTACCCGGCGCCTCCACCAAAATATAATTTGTTTTTTTATATTTTAGTGGGGGCGAAACAGGATCGACAAAAGTGTAAAGATTGCTCTTTTACTCGGTATAGTACCACCGTTATCGGACTAAATGAGTAGTTGCAAATGACAACTATGCGGAAGCACGTCTCGCTGCCTGAGGTGGTGTGAATGCTTCAAACTAAGTCTTAAACCGTCGCAGGTTTAAGCGGGGTTCGAAGGCACCTGGCAACAGAAGC | 298 bp |
|  | **Reverse primer**  **ssrA-R**  GCTTCTGTTGCCAGGTG |  |  |
| Neorickettsia | **Forward primer**  **groel-1500F**  ATAGATCCAGCKAAGGTAGTGCGTGT | ATAGATCCAGCTAAGGTAGTGCGTGTTGCCTTGGAGAGTGCTGTCTCTGTTGCTAGTGTTCTAGTGACTACCGAAGCCCTGATAGTTGATTTACCATCTAAAGATAACGGTTCTTCTTCAATGATGCCTGGTGGTGGCATGGGTGGAA | 148 bp |
|  | **Reverse primer**  **groel-1620R** TTCCACCCATGCCACCACCAGGCATCATTG |  |  |
| Rickettsia | **Forward primer**  **Ric16SF** TCCTAGTGTAGAGGTGAAATTCTTA | TCCTAGTGTAGAGGTGAAATTCTTAGATATTAGGAGGAACACCAGTGGCGAAGGCGGTCATCTGGGCTACAACTGACGCTGATGCACGAAAGCGTGGGGAGCAAACAGGATTAGATACCCTGGTAGTCCACGCCGTAAACGATGAGTGCTAGATATCGGAAGATTCTCTTTCGGTTTC | 178 bp |
|  | **Reverse primer**  **Ric16SR** GAAACCGAAAGAGAATCTTCCGAT |  |  |
| Anaplasma | **Forward primer**  **Ana/Ehr16SF** – ACACATGCAAGTCGAACG | AACACATGCAAGTCGAACGGATTTTTGTCGTAGCTTGCTATGATAAAAATTAGTGGCAGACGGGTGAGTAATGCATAGGAATCTACCTAGTAGTATGGGATAGCCACTAGAAATGGTGGGTAATACTGTATAATCCCTGCGGGGG | 145 bp |
|  | **Reverse primer**  **Ana/Ehr16SR** – CCCCCGCAGGGATTATACA |  |  |
| Ehrlichia | **Forward primer**  **Ehr16SF -** GGCACGTAGGTGGACTA | GGCACGTAGGTGGACTAGTAAGTTAAAAGTGAAATACCAAAGCTTAACTTTGGAGCGGCTTTTAATACTGCTAGACTAGAGGTCGAAAGAGGATAGCGGAA | 101 bp |
|  | **Reverse primer**  **Ehr16SR** - TTCCGCTATCCTCTTTCGAC |  |  |
| Borrelia | **Forward primer**  **BorP66F** - GCAATTTTAGCATCTTTTGGAG | GCAATTTTAGCATCTTTTGGAGCTCAATATAAGCTTGGATTAACAAAAATCAATGATAAAAATACTTATCTTATTTTGCAAATGGGAACTGATTTTGGAATAGATC | 106 bp |
|  | **Reverse primer**  **BorP66R** - GATCTATTCCAAAATCRGTWCC |  |  |
| Leishmania | **Forward primer**  **LEISH-1 -** AACTTTTCTGGTCCTCCGGGTAG | ACCCCCAGTTTCCCGCCCCGAACCCCAAAAATGGCAATTTTGGTCAAAAAATGAACGGGATTTCTGCACCCATTTTTCGGATTTCGCAGAACGCCCCTACCCGGAGGACCAGAAAAGTT | 119 bp |
|  | **Reverse primer**  **LEISH-2 -** ACCCCCAGTTTCCCGCC |  |  |

**Conventional PCR-based assays**

Conventional PCR-based assays were performed using the Biometra TAdvanced thermocycler (Analytic Jena). Primer concentrations were optimized for each conventional PCR-based pathogen assay. Details are shown in Supplemental Table 7.

**Supplemental Table 7.** Primer concentrations used in the conventional PCR-based assays

| Infectious agent | | Primer concentration | Amount used  in Master Mix |
| --- | --- | --- | --- |
| Bacteria | Leptospira interrogans | 5 pmol/µl | 1 µl |
|  | Brucella canis | 5 pmol/µl | 1 µl |
| Protozoa | Neospora | 5 pmol/µl | 1 µl |
|  | Babesia** | 2.5 pmol/µl | 4 µl |
| DNA viruses | CaV-1 (canine adenovirus) | 5 pmol/µl | 1.25 µl |
|  | CaHV-1 (canine herpesvirus) | 5 pmol/µl | 1.25 µl |
|  | CPV (canine parvovirus) | 5 pmol/µl | 1 µl |
|  | SuHV-1 (Suid herpesvirus 1) | 5 pmol/µl | 1.25 µl |
| RNA viruses | CIV (canine influenza virus) | 5 pmol/µl | 1 µl |
|  | CPIV (canine parainfluenza virus) | 5 pmol/µl | 1 µl |
|  | CDV (Canine distemper virus) | 5 pmol/µl | 1 µl |
|  | CcoV (Canine coronavirus) | 5 pmol/µl | 2.5 µl |
|  | Rabies virus | 5 pmol/µl | 1 µl |

*Leptospira interrogans* and *Brucella canis* detection

Endpoint PCR followed by agarose gel electrophoresis was used to detect *Leptospira interrogans* and *Brucella canis*.

The assay was carried out using the GoTaq DNA polymerase kit (Promega®). The PCR reaction mixture (25 µl) contained 5 μl of 5x Colorless GoTaq Flexi Buffer, 2 μl of 2.5 mM dNTP mix (Thermo Fisher Scientific), 2 µl of MgCl_2_ solution, 1 µl of the 5 μM primers each, 0.125 µl GoTaq® G2 Hot Start Polymerase, and 12.875 µl nuclease-free water; finally, 1 µl of isolated DNA was added to the mixture.

The PCR running conditions were 95ºC for 2 min followed by 45 cycles of 95ºC for 15 s, 56ºC for 30 s, and 72ºC for 30 s with a final extension at 72ºC for 5 min. The PCR reaction was performed using an Analytik Jena (Biometra Tadvanced) thermal cycler. PCR products were analyzed by agarose gel electrophoresis, using 2% agarose gel.

Specific primer sequences were used to detect the 16S rRNA gene for *Leptospira spp*. (M71241.1:581-751 *Leptospira interrogans* 16S ribosomal RNA) and the virB2 gene (AF226278.1: 1641-1921 *Brucella abortus*) for *Brucella spp*. Primers and target sequences are shown in Supplemental Table 8.

**Supplemental Table 8.** Primers and target sequences – *Leptospira interrogans* and *Brucella canis*

| Infectious agent | Primer name and sequence (5’-3’) | Target sequence (5’-3’) | Sequence length (bp) |
| --- | --- | --- | --- |
| *Leptospira interrogans* | **Forward primer**  **LepN-1** - CTGGCCTAAAACTGACGCTGA | CTGGCCTAAAACTGACGCTGAGGCACGAAAGCGTGGGTAGTGAACGGGATTAGATACCCCGGTAATCCACGCCCTAAACGTTGTCTACCAGTTGTTGGGGGTTTTNACCCTCAGTAACGAACCTNACGGATTNAGTAGACCGCCTGGGGACTATGCTCGCAAGAGTGAAAC | 171 bp |
|  | **Reverse primer**  **LepN-2** - CTTTCACTCTTGCGAGCATAG |  |  |
| *Brucella canis* | **Forward primer**  **B2N-1** – GTCGCGGATTCTACCTCACCT | GTCGCGGATTCTACCTCACCTACTGCTGGCCCTCATTGTCTCCATCGCTGCAATCGAGCCTAACCTGGCGCACGCCAACGGTGGCCTCGATAAGGTAAATACAAGCATGCAAAAAGTGCTGGACTTGCTAAGCGGCGTATCGATCACCATCGTTACCATAGCCATCATCTGGTCCGGTTACAAGATGGCATTCCGGCACGCCCGCTTCATGGATGTAGTGCCGGTGCTGGGCGGCGCCCTGGTGGTTGGCGCTGCCGCCGAAATTGCCTCTTACCTGCTTA | 281 bp |
|  | **Reverse primer**  **B2N-2** - TAAGCAGGTAAGAGGCAATTT |  |  |

Detection of DNA viruses

Endpoint PCR followed by agarose gel electrophoresis was used to detect DNA viruses Canine Adenovirus type-1 (CAV-1), Canine herpesvirus (CaHV-1), Canine parvovirus (CPV), and Suid herpesvirus-1 (SuHV1). These DNA viruses were tested using the same run protocol described below.

The assay was carried out using the GoTaq DNA polymerase kit (Promega®). The PCR reaction mixture (25 µl) contained 5 μl of 5x Colorless GoTaq Flexi Buffer, 2 μl of 2.5 mM dNTP mix (Thermo Fisher Scientific), 2 µl of MgCl_2_ solution, 1.25 µl (1 µl in the case of CPV) of the 5 μM primers each, 0.125 µl GoTaq® G2 Hot Start Polymerase, and 12.375 µl nuclease-free water; finally, 1 µl isolated DNA was added to the mixture.

The PCR running conditions were 94ºC for 5 min followed by 45 cycles of 95ºC for 30 s, 58ºC for 30 s, and 74ºC for 30 s with a final extension at 72ºC for 5 min. The PCR reaction was performed using an Analytik Jena (Biometra Tadvanced) thermal cycler. PCR products were analyzed by agarose gel electrophoresis, using 2% agarose gel.

Specific CAV-1 primers amplify a 545-bp fragment of the E3 gene (Y07760.1:24886-25430 Canine adenovirus type 1 complete genome), CaHV-1 primers a 136-bp fragment of the GB gene, CPV primers amplify a 337-bp partial sequence of the CPV genome, and SuHV1 primers amplify a 92-bp partial sequence of the SuHV-1 genome. Target sequences and specific primers are shown in Supplemental Table 9.

**Supplemental Table 9.** Primers and target sequences – DNA viruses

| Infectious agent | Primer name and sequence (5’-3’) | Target sequence (5’-3’) | Sequence length (bp |
| --- | --- | --- | --- |
| Canine adenovirus type‑1 (CAV‑1) | **Forward primer**  **CadV_E3_F25073**  TATTCCAGACTCTTACCAAGAGG | TATTCCAGACTCTTACCAAGAGGGACATGGTGTGAATGTGAAAATAACGTTTTCCCACCGCTCCAGAAACCTGCGCCACAATGGCCATGATGTAATATGTTCCTACTCACACCTGGGATCCCACATTAGCATAAGATGTACTTGTAACAAACCGCGTCCTCACCTAAGCCTAATTGAGGCGGCCTGTTCTATGTATAACCTTGACTAGATGTGATTAAACTTTTCTTGCAGCTACCGCGATAATGCGCTTCTGTTTCTTCTTCTGCTTCACCGCAAGCATTTTCTGCACTACAGGAAACAGCAGTGACATTGTATTTTGCTGCGCCCACACACCTTGCCTTCTACATCTAGAAGTGGACCAGGAAACCAGTGTCACTTGGATAGACTCTAACACAGGCCAAATTCCGCTCTGTCTCTCCAATGGCACATGCCACATAAGCGAAAAAGGCCTGCACTTTTCTGCAAATTTTTCCAAGGATGGCCTATACATCGCCATCATTAATGAAACAAACTATCATGCCGCTGAACATTACTACCTTGTCTAT | 545 bp |
|  | **Reverse primer**  **CadV_E3_R25623**  ATAGACAAGGTAGTARTGYTCAG |  |  |
| Canine herpesvirus (CaHV-1) | **Forward primer**  **CaHV_GBF439**  ACAGAGTTGATTGATAGAAGAGGTATG | ACAGAGTTGATTGATAGAAGAGGTATGTGTTTATCAAAAGCTGATTATATTCGTAATAATTATGAATTTACCGCATTTGATAAGGATGAAGACCCCAGAGAAGTTCATTTAAAGCCTTCAAAGTTTAATACACCAG | 136 bp |
|  | **Reverse primer**  **CaHV_GBR574**  CTGGTGTATTAAACTTTGAAGGCTTTA |  |  |
| Canine parvovirus (CPV) | **Forward primer**  **CPV-F**  AAGACGTGCAAGCGAGTCC | AAGACGTGCAAGCGAGTCCAACGTGGTCCGAAATAGAGGCAGACCTGAGAGCCATCTTTACTTCTGAACAATTGGAAGAAGATTTTCGAGACGACTTGGATTAAAGTACGATGGCACCTCCGGCAAAGAGAGCCAGGAGAGGTAAGGGTGTGTTAGTGAAGTGGGGGGAGGGGAAAGATTTAATAACTTAACTAAGTATGTATTTTTTTGTAGGACTTGTGCCTCCAGGTTATAAATATCTTGGGCCTGGGAACAGTCTTGACCAAGGAGAACCAACTAACCCTTCTGACGCCGCTGCAAAAGAACACGACGAAGCTTACGCTGCTTATCTTCGCTC | 337 bp |
|  | **Reverse primer**  **CPV-R**  GAGCGAAGATAAGCAGCGTAA |  |  |
| Suid herpesvirus 1 (SuHV1) | **Forward primer**  **gB-Taq-F**  CTCCTGCCGCACCTGAAG | GTCTGGAAGCGGTAGAAGCCGCGGAACTCGCTGACGCACCAATCGCGGGCCACCATGAAGCGCGCCAGCTCCTCCTTCAGGTGCGGCAGGAG | 92 bp |
|  | **Reverse primer**  **gB-Taq-R**  GTCTGGAAGCGGTAGAAGCC |  |  |

Detection of RNA viruses

Rabies virus (*Rabies lyssavirus*) detection

Hemi-nested PCR followed by agarose gel electrophoresis was used to detect Rabies virus. The assay was carried out using the GoTaq DNA polymerase kit (Promega®). The PCR reaction mixture (25 µl) contained 5 μl of 5x Colorless GoTaq Flexi Buffer, 2 μl of 2.5 mM dNTP mix (Thermo Fisher Scientific), 2 µl of MgCl_2_ solution, 1 µl of the 5 μM primers each, 0.125 µl GoTaq® G2 Hot Start Polymerase, and 12.875 µl nuclease-free water; finally, 1 µl isolated DNA was added to the mixture. In the second round PCR, 1 µl of the first round PCR product was used as template.

The PCR running conditions for the 1^st^ round were 95ºC for 5 min followed by 45 cycles of 94ºC for 30 s, 45ºC for 45 s, 50ºC for 15 s, and 72ºC for 1 min with a final extension at 72ºC for 7 min. The PCR running conditions for the 2^nd^ round PCR were 95ºC for 5 min followed by 35 cycles of 94ºC for 30 s, 45ºC for 10 s, 50ºC for 15 s, and 72ºC for 1 min with a final extension at 72ºC for 7 minutes. The PCR reactions were performed using an Analytik Jena (Biometra Tadvanced) thermal cycler. The PCR products were analyzed by agarose gel electrophoresis, using 2% agarose gel.

The assay targets the N gene (part of the nucleoprotein) of lyssaviruses, which is a conserved region of the genome. The 1^st^ round PCR uses the universal primers JW12 (forward) and JW6UNI (reverse) to detect a 606-bp region of the N gene. The reverse primer JW10UNI, which targets a sequence of the 1^st^ PCR product, is used in conjunction with the JW12 primer in the second round of the hemi-nested PCR and amplifies a 582-bp sequence. 1 µl of the 1^st^ round PCR product is used in the 2^nd^ PCR. The target sequence and specific primers are shown in Supplemental Table 10.

**Supplemental Table 10.** Primers and target sequence – Rabies virus

| Infectious agent | Primer name and sequence (5’-3’) | Target sequence (5’-3’) | Sequence length (bp) |
| --- | --- | --- | --- |
| Rabies virus | **Forward primer**  **JW12-F**  ATGTAACACCYCTACAATG | ATGTAACACCCCTACAATGGATGCCGACAAGATTGTATTCAAAGTTAATAATCAGGTGGTCTCTTTGAAGCCTGAGATTATCGTGGATCAATATGAGTACAAGTACCCTGCTATTAAGGACTTGAAGAAGCCCAGTATCACCCTAGGTAAGGCCCCTGATTTGAACAAGGCATACAAGTCAGTTTTATCAGGCTTGAATGCTGCCAAGCTTGATCCTGATGACGTATGTTCCTATTTAGCAGCCGCAATGCAGTTCTTCGAGGGGACGTGTCCCGAAGACTGGACCAGCTATGGGATCCTGATTGCACGGAAAGGAGATAAGATCACCCCAGATTCTCTGGTGGAGATAAAGCGTACTGGTGTAGAAGGGAATTGGGCTTTGACGGGAGGGATGGAACTGACTAGGGACCCCACTGTTCCAGAGCATGCGTCTCTAGTCGGTCTTCTCTTGAGTCTGTATAGACTAAGCAAAATATCTGGGCAAAACACTGGTAACTATAAGACAAACATTGCAGATAGGATAGAGCAGATTTTCGAGACAGCCCCCTTTATTAAGATCGTGGAGCATCATACTCTGATGAC | 582 bp |
|  | **Reverse primer**  **JW6 UNI-R**  ARTTVGCRCACATYTTRTG |  |  |
|  | **Reverse primer**  **JW 10 UNI-R**  GTCATYARWGTRTGRTGYTC |  |  |

Canine coronavirus (CcoV) detection

Endpoint PCR followed by agarose gel electrophoresis was used to detect canine coronavirus. The assay was carried out using the GoTaq DNA polymerase kit (Promega®). The PCR reaction mixture (25 µl) contained 5 μl of 5x Colorless GoTaq Flexi Buffer, 2 μl of 2.5 mM dNTP mix (Thermo Fisher Scientific), 3 µl of MgCl_2_ solution, 2.5 µl of the 5 μM primers each, 0.125 µl GoTaq® G2 Hot Start Polymerase, and 8.875 µl nuclease-free water; finally, 1 µl isolated DNA was added to the mixture.

The PCR running conditions were 95ºC for 1 min followed by 45 cycles of 95ºC for 15 s, 58ºC for 30 s, and 72ºC for 30 s with a final extension at 72ºC for 5 min. The PCR reaction was performed using an Analytik Jena (Biometra Tadvanced) thermal cycler. The PCR products were analyzed by agarose gel electrophoresis, using 2% agarose gel.

Specific primers were used to detect a 452-bp sequence of the canine coronavirus (Canine coronavirus strain CB/05, KP981644.1:14134-14672). The target sequence and the specific primers are shown in Supplemental Table 11.

**Supplemental Table 11.** Primers and target sequence – Canine coronavirus

| Infectious agent | Primer name and sequence (5’-3’) | Target sequence (5’-3’) | Sequence length (bp) |
| --- | --- | --- | --- |
| Canine coronavirus | **Forward primer**  **CoV_16053_F** GGTTGGGAYTAYCCTAARTGTGA | GGATGGGACTATCCTAAGTGTGACCGCGCTTTACCTAATATGATCAGAATGGCATCTGCCATGATATTAG  GTTCTAAGCACGTTGGTTGTTGTACACATAGTGATAGATTCTACCGTCTCTCCAATGAGTTAGCTCAAGT  ACTCACAGAAGTCGTGCATTGCACAGGTGGGTTTTACTTTAAACCTGGTGGTACAACTAGCGGTGATGGT  ACTACAGCTTATGCTAACTCAGCTTTTAACATCTTTCAAGCTGTTTCTGCTAATGTTAATAAGCTTTTGG  GAGTCGATTCAAACGCTTGTAACAATGTTACAGTAAAATCTATACAACGTAAAATCTACGATAATTGTTA  TCGCAGTAGCAGCATTGATGAAGAATTTGTTGTTGAGTACTTTAGTTATTTGAGAAAACACTTTTCTATG  ATGATTTTGTCTGATGATGGAGTTGTGTGCTACAA | 452 bp |
|  | **Reverse primer**  **CoV_Pan_16510**  TTATARCAVACAACNCCATCATCA |  |  |

Canine influenza virus (CIV), canine parainfluenza virus (CPIV) and canine distemper virus (CDV) detection

Endpoint PCR followed by agarose gel electrophoresis was used to detect Canine infectious respiratory disease complex (CIRDC) viruses, canine influenza virus (CIV), canine parainfluenza virus (CPIV), and canine distemper virus (CDV). These viruses were tested with the same run protocol. The PCR reaction mixture (25 µl) contained 5 μl of 5x Colorless GoTaq Flexi Buffer, 2 μl of 2.5 mM dNTP mix (Thermo Fisher Scientific), 3 µl of MgCl_2_ solution, 1 µl of the 5 μM primers each, 0.125 µl GoTaq® G2 Hot Start Polymerase, and 11.875 µl nuclease-free water; finally, 1 µl isolated DNA was added to the mixture.

The PCR running conditions were 94ºC for 5 min followed by 45 cycles of 95ºC for 30 s, 55ºC for 30 s, and 74ºC for 30 s with a final extension at 72ºC for 5 min. The PCR reaction was performed using an Analytik Jena (Biometra Tadvanced) thermal cycler. PCR products were analyzed by agarose gel electrophoresis, using 2% agarose gel.

CIV primers amplify a 126-bp fragment of the M gene, CPIV primers a 186-bp fragment of the NP gene (MH727491.1 Canine parainfluenza virus strain CPIV-J2 nucleocapsid protein N gene), and CDV primers a 290-bp fragment of the NP gene. Target sequences and specific primers are shown in Supplemental Table 12.

**Supplemental Table 12.** Primers and target sequences – Canine infectious respiratory disease complex (CIRDC) viruses

| Infectious agent | Primer name and sequence (5’-3’) | Target sequence (5’-3’) | Sequence length (bp) |
| --- | --- | --- | --- |
| CIV | **Forward primer**  **CIV_M_F151**  CATGGARTGGCTAAAGACAAGACC | CATGGAATGGCTAAAGACAAGACCAATCCTGTCACCTCTGACTAAAGGGATTTTAGGATTTGTATTCACGCTCACCGTGCCCAGTGAGCGAGGACTGCAGCGTAGACGCTTTGTCCAAAATGCCCT | 126 bp |
|  | **Reverse primer**  **CIV_M_R276**  AGGGCATTTTGGACAAAKCGTCTA |  |  |
| CPIV | **Forward primer**  **CPIV_N_F428**  GCCGTGGAGAGATCAATGCCTAT | GCCGTGGAGAGATCAATGCCTATGCTGCACTTGCAG  AAGATCTACCTGACACACTAAACCATGCAACACCTTTCGTTGATTCCGAAGTCGAGGGAACTGCATGGGATGAGATTGAGACTTTCTTAGATATGTGTTACAGTGTCCTAATGCAGGCATGGATAGTGACTTGCAAGTGCATGACTGCGC | 186 bp |
|  | **Reverse primer**  **CPIV_N_R614**  GCGCAGTCATGCACTTGCAAGT |  |  |
| CDV | **Forward primer**  **CDV_N_F768**  AACAGRRATTGCTGAGGACYTAT | AACAGGATTGCTGAGGACCTATCTTTGAGGCGATTCATGGTGGCGCTCATCTTGGACATCAAACGATCCCCAGGGAACAAGCCTAGAATTGCTGAAATGATTTGTGATATAGATAACTACATTGTGGAAGCTGGGTTAGCTAGTTTCATCCTAACTATCAAGTTTGGCATTGAAACTATGTATCCGGCTCTTGGGTTGCATGAGTTTTCCGGAGAATTAACAACTATTGAATCCCTCATGATGCTATATCAACAGATGGGTGAAACAGCACCGTACATGGTTATCTTGGA | 290 bp |
|  | **Reverse primer**  **CDV_N_R1057** TCCARRRATAACCATGTAYGGTGC |  |  |

*Neospora caninum* detection

Endpoint PCR followed by agarose gel electrophoresis was used to detect *Neospora caninium*. The PCR reaction mixture (25 µl) contained 5 μl of 5x Colorless GoTaq Flexi Buffer, 2 μl of 2.5 mM dNTP mix (Thermo Fisher Scientific), 3 µl of MgCl_2_ solution, 1 µl of the 5 μM primers each, 0.125 µl GoTaq® G2 Hot Start Polymerase, and 11.875 µl nuclease-free water; finally, 1 µl of isolated DNA was added to the mixture.

The PCR running conditions were 95ºC for 2 min, 10 cycles of 94ºC for 30 s, 57ºC for 30 s, 72ºC for 20 s; 30 cycles of 86ºC for 30 s, 54ºC for 30 s,72ºC for 20 s, and 72ºC for 2 minutes. The PCR reaction was performed using an Analytik Jena (Biometra Tadvanced) thermal cycler. The PCR products were analyzed by agarose gel electrophoresis, using 2% agarose.

Specific primer sequences were used to detect a 146-bp fragment of internal transcribed spacer 1 region of ribosomal DNA of *Neospora caninum.* Specific primers and the target sequence are shown in Supplemental Table 13.

**Supplemental Table 13**. Primers and target sequence – *Neospora caninum*

| Infectious agent | Primer name and sequence (5’-3’) | Target sequence (5’-3’) | Sequence length (bp) |
| --- | --- | --- | --- |
| *Neospora caninum* | **Forward primer**  NS2 – CATGTGGATATTTTGCA | CATGTGGATATTTTGCACTACTTTTTTCAAGCGTTCTATTGAACGCCTGATAATGAAAGTGTGTGCATATATCCGGGAGTGTACGGCGAAGGGACTCGGTCACTGGAAATTAATGTCTCTATTGGGACTTTAACTTCCAGGAGTTT | 146 bp |
|  | **Reverse primer**  NR1 - AAACTCCTGGAAGTTAAAG |  |  |

Babesia detection

Single tube multiplex PCR followed by agarose gel electrophoresis was used to detect Babesia species. The PCR reaction mixture (25 µl) contained 5 μl of 5x Colorless GoTaq Flexi Buffer, 2 μl of 2.5 mM dNTP mix (Thermo Fisher Scientific), 3 µl of MgCl_2_ solution, 4 µl of the primer pool containing the 3 different forward primers (2.5 µM each) and a single reverse primer, 0.125 µl GoTaq® G2 Hot Start Polymerase and 9.875 µl nuclease-free water, and finally 1 µl of isolated DNA was added to the mixture.

The PCR running conditions were the following: 95 °C for 2 min followed by 40 cycles of 95 °C for 15 s, touchdown from 63°C to 55 °C for 30 s (gradual reduction of the temperature by 0.2 °C in every consecutive cycle); and 72 °C for 30 s with a final extension at 72ºC for 2 min. The PCR reaction was performed using an Analytik Jena (Biometra Tadvanced) thermal cycler. PCR products were analyzed by agarose gel electrophoresis, using 2% agarose gel.

Specific forward primers B-BM, B-BDV, and B-BDV2 and reverse primer B-rev (Supplemental Table 14) amplify 381-bp and 408-bp 18S rRNA gene fragments of *B. divergens*, *B. divergens-like*, *B. venatorum, B. microti*, *Babesia microti-like* and *Babesia canis*.

**Supplemental Table 14**. Primers and target sequence – Babesia

| Infectious agent | Primer name and sequence (5’-3’) | Target sequence (5’-3’) | Sequence length (bp) |
| --- | --- | --- | --- |
| Babesia | **Forward primer**  **B-BM** - GAATCTAAACCCTTCCCAGAGTATC | TGACCTAAACCCTCACCAGAGTAACAATTGGAGGGCAAGTCTGGTGCCAGCAGCCGCGGTAATTCCAGCTCCAATAGCGTATATTAAACTTGTTGCAGTTAAAAAGCTCGTAGTTGAATTTTTGCGTGGTGTTAATATTGACTGATGTCGAGATTGCACTTCTCTTTTGGGATTTATCCCTTTTTACTTTGATAAAATTAGAGTGTTTCAAGCAGACTTTTGTCTTGAATACTTCAGCATGGAATAATAGAGTAGGACTTTGGTTCTATTTTGTTGGTTTGTGAACCTTAGTAATGGTTAATAGGAACGGTTGGGGGCATTCGTATTTAACTGTCAGAGGTGAAATTCTTAGATTTGTTAAAGACGAACTACTGCGAAAGC | 381 bp |
|  | **Forward primer**  **B-BDV** - TGACCTAAACCCTCACCAGAGTAAC |  |  |
|  | **Forward primer**  **B-BDV2** - TGACCCAAACCCTCACCAGAGTARC |  |  |
|  | **Reverse primer**  **B-rev** - GCTTTCGCAGTAGTTCGTCTTTA |  |  |
